# Bumblebees compensate for the adverse effects of sidewind during visually guided landings

**DOI:** 10.1242/jeb.245432

**Published:** 2024-04-22

**Authors:** Pulkit Goyal, Johan L. van Leeuwen, Florian T. Muijres

**Affiliations:** Experimental Zoology Group, Wageningen University and Research, 6708 WD Wageningen, The Netherlands

**Keywords:** *Bombus terrestris*, Biomechanics, Control theory, Insect flight, Maneuverability, Sensorimotor control

## Abstract

Flying animals often encounter winds during visually guided landings. However, how winds affect their flight control strategy during landing is unknown. Here, we investigated how sidewind affects the landing performance and sensorimotor control of foraging bumblebees (*Bombus terrestris*). We trained bumblebees to forage in a wind tunnel, and used high-speed stereoscopic videography to record 19,421 landing maneuvers in six sidewind speeds (0 to 3.4 m s^−1^), which correspond to winds encountered in nature. Bumblebees landed less often in higher windspeeds, but the landing durations from free flight were not increased by wind. By testing how bumblebees adjusted their landing control to compensate for adverse effects of sidewind on landing, we showed that the landing strategy in sidewind resembled that in still air, but with important adaptations. Bumblebees landing in a sidewind tended to drift downwind, which they controlled for by performing more hover maneuvers. Surprisingly, the increased hover prevalence did not increase the duration of free-flight landing maneuvers, as these bumblebees flew faster towards the landing platform outside the hover phases. Hence, by alternating these two flight modes along their flight path, free-flying bumblebees negated the adverse effects of high windspeeds on landing duration. Using control theory, we hypothesize that bumblebees achieve this by integrating a combination of direct aerodynamic feedback and a wind-mediated mechanosensory feedback control, with their vision-based sensorimotor control loop. The revealed landing strategy may be commonly used by insects landing in windy conditions, and may inspire the development of landing control strategies onboard autonomously flying robots.

## Teaser

**Highlighted Article:** Bumblebees foraging in strong sidewinds can still land precisely on artificial flowers, allowing them to be efficacious and robust pollinators in these adverse environmental conditions.

## INTRODUCTION

Wind is an important characteristic of the natural world that affects both ecological interactions and the biomechanics of flying insects. Wind affects their migration and dispersal (Hu et al., 2016a,b; Leitch et al., 2021; Mikkola, 1986; Pasek, 1988), interaction with plants and flowers (Alcorn et al., 2012; Alma et al., 2017), and floral visitation rates (Crall et al., 2020; Hennessy et al., 2021). Wind imposes maneuverability challenges (Burnett et al., 2020; Chang et al., 2016; Jakobi et al., 2018; Matthews and Sponberg, 2018; Mountcastle et al., 2015; Ortega-Jimenez et al., 2013, 2014; Ravi et al., 2013, 2016) that may increase the energetic costs of flight (Combes and Dudley, 2009; Crall et al., 2017). Unraveling how flying insects cope with the effects of winds can help us to understand their neuroethology, biomechanics and ecology, as well as provide guiding principles for the development of wind mitigation strategies in manufactured aerial vehicles.

Landing is an important behavior for all flying animals, in particular for animals such as bumblebees that rely on their landing ability to gather food essential for survival and reproduction (Goulson, 2010; Michener, 2007). Successful landing requires precise control of flight speed as an animal approaches the surface (Baird et al., 2013; Goyal et al., 2021; Srinivasan et al., 2000). While visiting flowers, foraging bumblebees land very frequently, with up to a thousand landings in an hour (Heinrich, 1979; Michener, 1974), and often in a wide range of wind conditions (Crall et al., 2017; Peat and Goulson, 2005; Riley et al., 1999).

**Fig. 1.**
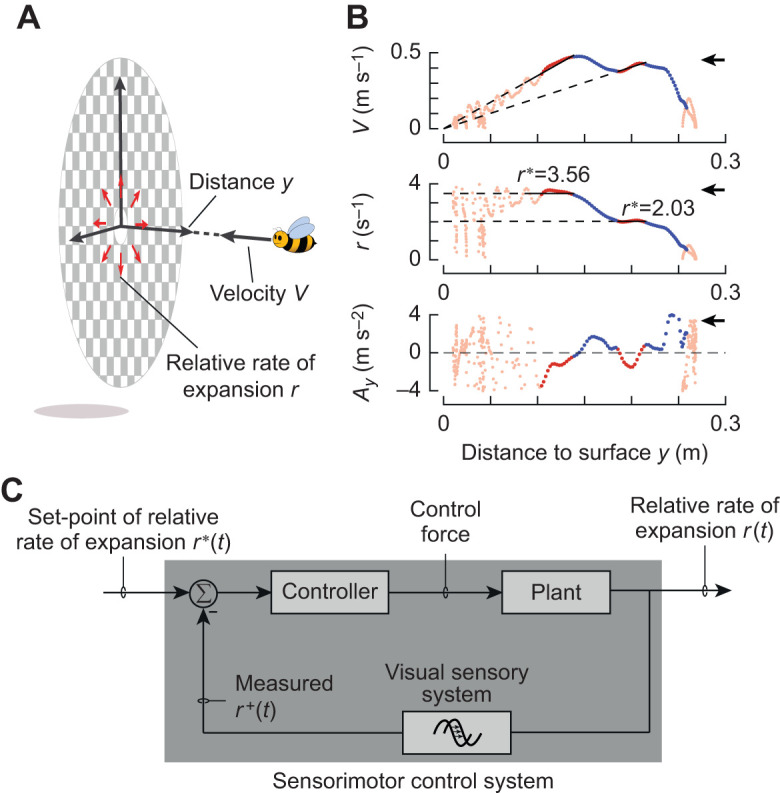
**Visually guided landing strategy and proposed sensorimotor control system of flying bumblebees.** (A) Diagram of a bumblebee flying with perpendicular approach velocity *V* towards a landing platform. At a distance *y*, it experiences a relative rate of optical expansion *r*=*V*/*y*. (B) The bumblebee landing kinematics with plots of *V*–*y* (top), *r*–*y* (middle) and approach acceleration *A_y_*=d*V*/d*t* versus *y* (bottom). The approach consists of an alternating series of entry segments (blue lines) and constant-*r* segments (red lines). Corresponding estimated set-points r* are depicted by dashed black lines as slope and ordinate values in the *V*–*y* and *r*–*y* graphs, respectively. The black arrows indicate flight direction towards the landing surface. (C) Proposed closed-loop sensorimotor control system that landing bumblebees use to converge the optical expansion rate *r* to a set-point r*. Using their visual system, bumblebees measure optical expansion rate as *r*^+^. Based on the difference between *r*^+^ and r*, the animal produces a proportional aerodynamic control force, which accelerates the animal (‘plant’ in control terminology).

In the absence of wind, many flying animals – including bumblebees – use visual feedback to control their flight speed as they advance towards the landing surface and achieve a soft touchdown (Baird et al., 2013; Balebail et al., 2019; Chang et al., 2016; Goyal et al., 2021, 2023; Lee et al., 1991, 1993; Liu et al., 2019; Reber et al., 2016; Shackleton et al., 2019; Srinivasan et al., 2000; Tichit et al., 2020; Van Breugel and Dickinson, 2012; Whitehead, 2023). Their motion relative to the landing surface generates optical expansion cues in which various features in the visual image appear to move radially outward from the point that is being approached (Edwards and Ibbotson, 2007; Gibson, 1955). Bumblebees can use this optical flow relative to the retinal image size of an object (Wagner, 1982), or the angular position of features in the image (Baird et al., 2013), to measure optical expansion rate, also known as relative rate of expansion. The instantaneous optical expansion rate *r* is equal to the ratio of approach velocity *V* of the bumblebee and its distance from the surface *y* (*r*=*V*/*y*; Fig. 1A). Bumblebees use this optical expansion rate to control their landing (Chang et al., 2016; Goyal et al., 2021).

When doing so, a bumblebee approaches a landing surface in still air using a series of approach bouts (Fig. 1B) (Goyal et al., 2021). During each bout, a bumblebee regulates the optical expansion rate *r*, and uses its sensorimotor control system to produce the motor output needed to reach a particular value of the optical expansion rate, also known as an optical expansion rate set-point r* (Fig. 1C) (Baird et al., 2013). As a result of these control actions, each flight bout consists of two phases (Fig. 1B) (Goyal et al., 2022). During the so-called constant-*r* phase, the bumblebee flies approximately at the optic expansion rate set-point, as regulated using steady-state control responses. Preceding the constant-*r* phase, bumblebees tend to use their transient flight responses to converge towards the constant-*r* set-points; we call these flight phases ‘entry segments’. From one bout to the next, bumblebees tend to increase their set-point in a stepwise manner, leading to a new entry segment followed by a constant-*r* flight phase.

This stepwise upregulation of the optic expansion set-points causes landing bumblebees to intermittently increase their approach flight speed during the entry segments, and decrease it during the constant-*r* flight phase (Goyal et al., 2022). In addition to these accelerations and decelerations, bumblebees occasionally exhibit low-velocity phases, also described as hover phases (de Vries et al., 2020; Goyal et al., 2021; Reber et al., 2016). These may result from an instability resulting from a flight controller that uses optical expansion rate as a control variable (de Croon, 2016).

During landing in wind, bumblebees experience different airspeeds around their wings and body as compared with those in still air. As this airspeed influences the aerodynamic forces and torques that bumblebees produce with their flapping wings, it becomes mandatory for the bumblebees to adapt their sensorimotor control response for successful landings. This adaptation can be based on the measurement of airspeed, possibly with their antennae (Jakobi et al., 2018; Taylor and Krapp, 2007), and must generate forces and torques that compensate for the effects of winds (Dickinson and Muijres, 2016; Dickinson et al., 2000).

In nature, winds are often characterized by a mean wind and the fluctuations around it (Garratt, 1994; Stull, 1988). Although the effects of mean wind and wind fluctuations on the locomotory performance have been studied in freely flying insects (Baird et al., 2021; Barron and Srinivasan, 2006; Burnett et al., 2022; Crall et al., 2017; Engels et al., 2016; Fuller et al., 2014; Laurent et al., 2021; Ravi et al., 2015; Shepard et al., 2016), their effects on the landing behavior have received little attention. To our knowledge, only one study suggests that winds influence the landing dynamics of bumblebees (Chang et al., 2016), but it is unknown how bumblebees achieve flight control during these landings in winds.

To address this knowledge gap, we investigated the landing dynamics of bumblebees in the presence of various levels of steady sidewinds. Specifically, we studied how a steady sidewind affects the vision-based modular guidance strategy and the sensorimotor control of landing bumblebees, and how bumblebees cope with these, potentially detrimental, effects. For this purpose, we exposed foraging bumblebees to six different steady horizontal winds ranging from 0 to 3.4 m s^−1^, directed parallel to the landing surface. These conditions correspond to the typical wind speeds that bumblebees experience in nature (Crall et al., 2017). Moreover, we applied sidewinds as bumblebees often encounter crosswinds during flight (Riley et al., 1999), and flying insects, including bumblebees, are most sensitive to the aerial disturbances along the lateral axis (Ravi et al., 2013, 2016; Vance et al., 2013).

**List of symbols**

*a*: shape scale parameter in gamma distribution
**A**=(*a_x_,a_y_,a_z_*): body acceleration vector in the *x*,*y*,*z* coordinate system
*A_x_*: streamwise acceleration of bumblebee relative to ground: *A_x_*(*t*)=*a_x_*(*t*)
*A_y_*: approach acceleration of bumblebee: *A_y_*(*t*)=−*a_y_*(*t*)
*A_z_*: vertical acceleration of bumblebee: *A_z_*(*t*)=*a_z_*(*t*)
A*: state variable: A*={A_x_^*^, A_y_^*^, A_z_^*^}; mean value of {*A_x_*, *A_y_*, *A_z_*} within the constant-*r* phase
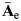
mean body acceleration during entry segment: 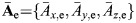
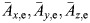
mean sideways, normal to platform and upward body accelerations during entry phase
*b*: inverse scale parameter in gamma distribution
*c*: intercept in linear regression
*f*: sensitivity factor used to select constant-*r* phases in flight tracks
*N*: number of landings per hour
*N*_freeflight,*i*,*d*,*t*_: number of landing maneuvers per hour for landing from free-flight, for the *i*th measurement on the *d*th day at the *t*th time (Eqn 1)
*N*_takeoff,*i*,*d*,*t*_: number of landing maneuvers per hour for landing after take-off, for the *i*th measurement on the *d*th day at the *t*th time (Eqn 1)
*m*: slope of a linear regression between the log transformations of r* and y* during constant-*r* phases
*P*_hover_: probability of the bumblebee exhibiting a hover phase
*r*: instantaneous optical expansion rate: *r*(*t*)=*V*(*t*)/*y*(*t*)
*r*^+^: optical expansion rate measured by the bumblebee
r*: set-point of relative optical expansion rate
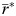
mean value of r*
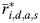
mean relative rate of expansion for *i*th measurement from *d*th day (*d*∈{1*,*2*,…,*11}), *a*th measured landing approach (*a*∈{1,2,…,19421}) and landing side *s* (Eqns 2 and 3)
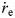
relative optical expansion acceleration during an entry segment
*t*: time relative to touchdown (*t*=0 s at touchdown)
**U**_A_: velocity of bumblebee relative to air (air speed; ignoring effect of bumblebee on air flow): **U**_A_=**U**_G_–**U**_W_=(*u*_A_,*v*_A_,*w*_A_)=(*u*_G_−*u*_W_,*v*_G_,*w*_G_)
*U*_A_: airspeed, magnitude of **U**_A_
**U**_G_=(*u*_G_,*v*_G_,*w*_G_): velocity of bumblebee in the *x*,*y*,*z* coordinate system (ground velocity)
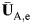
mean air velocity of the bumblebee during an entry segment: 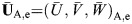
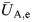
mean airspeed during an entry segment
*U*: streamwise speed of bumblebee relative to the ground: *U*(*t*)=*u*_G_(*t*)
**U**_W_=(*u*_W_,0,0): wind velocity; *U*_W_=*u*_W_ is wind speed
U*: state variable: U*={U*,V*,W*}; mean value of {*U*,*V*,*W*} in the constant-*r* flight phase
*V*: approach velocity of bumblebee perpendicular to platform, *V*(*t*)=−*v*_G_(*t*)
*W*: vertical velocity of bumblebee: *W*(*t*)=*w*_G_(*t*)
WIND*_j_*_,*i*,*d*,*t*_: indicates whether *j*th wind condition is present for the *i*th measurement on the *d*th day, and *t*th timeslot (0=no, 1=yes) (Eqn 1)
WIND*_j_*_,*i*,*d*,*a*,*s*_: indicates whether *j*th wind condition is present for *i*th measurement, from *d*th day, *a*th landing and landing side *s* (0=no, 1=yes) (Eqns 2 and 3)
**X**: space–time array: **X**=(*x*,*y*,*z*,*t*)
X*: state variable: X*={x*,y*,z*}; mean value of {*x*,*y*,*z*} during the constant-*r* phase
**X**_0_: position of bumblebee at start of entry segment: **X**_0_={*x*_0_,*y*_0_,*z*_0_}
*x*,*y*,*z*: Cartesian coordinate system, with *x* pointing in downstream direction, *y* is normal to landing platform directed into tunnel, and *z* pointing vertically upward
*x*_0_: streamwise position of the bumblebee at the start of an entry segment
*y*_0_: distance of bumblebee to landing surface at the start of an entry segment
*y*_1_,*y*_2_,*y*_3_,*y*_4_: coordinates used to distinguish sections relative to landing platform: 0.05<*y*_1_<0.10 m, 0.10<*y*_2_<0.15 m, 0.15<*y*_3_<0.20 m and 0.20<*y*_4_<0.25 m
y*_i__,_*_d_*_,_*_a_*_,_*_s_*: mean distance from platform during constant-*r* phase, for the *i*th measurement on *d*th day, for *a*th landing and at landing side *s* (Eqn 3)
*z*_0_: height of the bumblebee at the start of an entry segment
α: regression intercept for zero wind speed (Eqns 1 and 2)
α*_a_*: landing-approach-specific intercept (Eqn 2)
α*_d_*: day-specific regression intercept (Eqns 1 and 2)
α*_s_*: landing-side-specific intercept (Eqn 2)
α*_t_*: time-slot-specific regression intercept (Eqn 1)
β*_j_*: differences of fixed-effects (wind conditions) from overall intercept: β*_j_*∀*j*∈{1,2,…,5} (Eqns 1 and 2)
Δ*r*_e_: change in relative optical expansion rate during entry segment
Δ*r*: step in relative optical expansion rate between constant-*r* phases
Δr*: step in set-point of *r*
Δ*t*: travel time during the landing approach
ε: residual used in linear regression
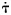
time-to-contact rate for landing of birds
σ: (residual) standard deviation (Eqn 1)

## MATERIALS AND METHODS

### Experimental animals, setup and procedure

During our indoor experiments, lab temperature was maintained at 21±2°C. We used a hive with more than 50 female worker bumblebees [*Bombus terrestris* (Linnaeus 1758)], provided by Koppert BV (Berkel en Rodenrijs, The Netherlands). The worker bees needed to perform forager flights to gather food from a 50% sugar solution, whereas dried pollen was provided in the hive.

**Fig. 2.**
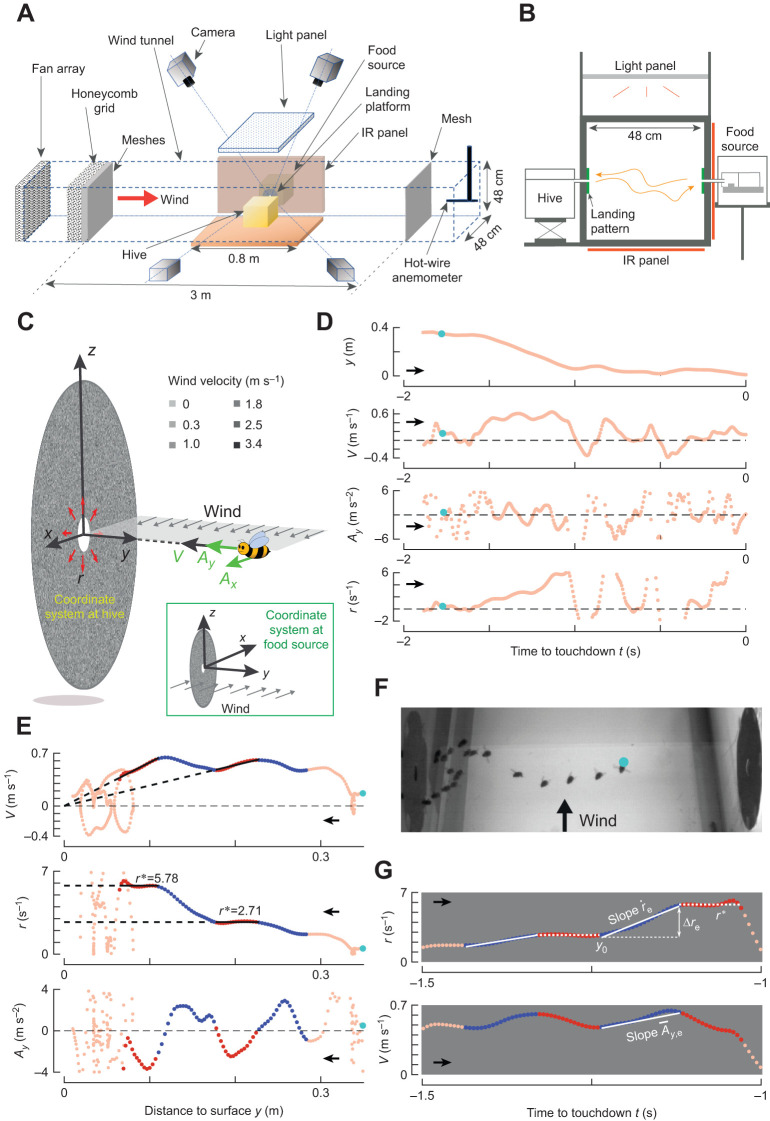
**Experimental setup, flight kinematics during landing and definitions of parameters.** (A,B) The experimental setup consisted of a wind tunnel with two vertically placed circular landing surfaces, one connected to a hive and one to a food source. Foraging bumblebees that flew between these landing surfaces were tracked in real time using a four-camera videography system. Visible and IR LED light panels were used for background illumination and videography illumination, respectively. (C) Landings on each platform were quantified in Cartesian coordinate systems, where the *x*-axis is parallel to the wind direction. (D–F) Landing maneuver of a free-flying bumblebee in a 3.4 m s^−1^ sidewind. In all panels, cyan dots denote the same instance, and black arrows indicate flight direction towards the landing surface (opposite in D and E). Parameters are approach distance *y*, velocity *V*, acceleration *A_y_* and relative rate of optical expansion *r*=*V*/*y*. (D) Temporal dynamics of (*y*,*V*,*A_y_*,*r*), with *t*=0 s at touchdown. (E) Variation of (*V*,*r*,*A_y_*) with *y*, where the constant-*r* and entry segments are in blue and red, respectively. (F) Photomontage from a top-view video at a time interval of ∼0.1 s. (G) Definition of entry segment parameters: optical expansion acceleration (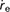), required step-change in optical expansion rate (Δ*r*_e_), associated set-point (r*), initial approach distance at entry segment start (*y*_0_) and average acceleration (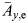).

Our experimental setup consisted of a wind tunnel (3.0×0.5×0.5 m; length×width×height), the bumblebee hive, a food source and a real-time machine-vision-based videography system (Fig. 2A). The hive and the food source were placed on two opposite sides of the middle section of the wind tunnel. Both were connected to the vertical walls of the wind tunnel (transparent polycarbonate 0.01 m thick sheets) using Plexiglass tubes (0.02 m diameter). These tubes were flush with the inside of the vertical tunnel walls. We attached circular landing platforms (0.18 m diameter) around each opening, with a visual pattern consisting of squares (1×1 mm) filled with random grayscale values (Fig. 2B). The setup was illuminated with a white broad-spectrum LED light panel to produce a light intensity similar to overcast daylight conditions (1823 lx; for details, see Goyal et al., 2021).

To generate different steady wind conditions in the wind tunnel, we built two 6×6 grids of DC cooling fans (San Ace 80 9GA0812P7S001 or 9GV0812P4K03, Sanyo Denki Co., Japan). Both grids were powered with a 480 W power supply (Mean Well SP-480-12, Mean Well Co., Taiwan). The air flow generated by a fan-grid traveled through a honeycomb structure (Tubes core PC, diameter 6 mm, 100 mm thickness, Tubus Bauer GmbH, Germany) and a sequence of four meshes (FG1814F fiberglass mosquito netting, 1.17×1.59 mm aperture, 68% transparency, Wire Waving Dinxperlo, The Netherlands) before it reached the wind tunnel test section. This was done to break down the fan-generated vortices and produce a low-turbulence uniform airflow.

We characterized the air flow in the wind tunnel using a hot-wire CTA anemometer (Dantec 55P16 wire probe and 54T42 MiniCTA, Dantec Dynamics, Denmark). We systematically measured the wind speed at various fan settings, days and locations within the tunnel. Firstly, we quantified the airflow and the deviations in its uniformity in the wind tunnel cross-section, in the middle of the wind tunnel, and at 0.20 m downstream and upstream locations. This showed that up to 4 cm from the walls, the airflow speeds remain within 94% of the mean windspeed. Secondly, we measured the airflow variation over time for 11 consecutive days of experiments. Within this period, the windspeed varied maximally 2% from the mean windspeed. Thirdly, we used the hot-wire anemometer to quantify the turbulence intensity (standard deviation of the airflow speed divided by the mean) of the airflow in our setup. This was less than 3% for all measured locations and for all tested wind speeds. These tests indicate that bumblebees experienced low-turbulent and close to uniform wind conditions at distances of more than 0.04 m from the walls.

We investigated the landing dynamics of bumblebees in zero wind, and in five steady wind speeds of 0.3, 1.0, 1.8, 2.5 and 3.4 m s^−1^. These winds span the range of speeds that bumblebees commonly experience in nature (Crall et al., 2017). To generate these wind conditions, we controlled either of the two fan-grids using pulse-width modulation (the San Ace 80 9GA0812P7S001 fan-grid for 0.3 and 1.0 m s^−1^ winds, and the San Ace 80 9GV0812P4K03 fan-grid for 1.8, 2.5 and 3.4 m s^−1^ winds). For the zero-wind condition, the fans were turned off.

Before starting the experiments, we trained the hive for 4 days to forage in still air. During training and experiments, bumblebees experienced a 10 h:14 h day:night cycle. Each day, sunrise was simulated between 07:30 h and 08:00 h by gradually increasing the light intensity from 0 to 1823 lx. Between 17:00 h and 17:30 h, we simulated sunset by gradually decreasing the light intensity back to 0 lx. During these timeslots, bumblebees were exposed to the zero-wind condition. We divided the rest of the day (08:00–17:00 h) into six 1.5-h timeslots, and bumblebees were exposed to one wind condition in each timeslot following a pseudo-random schedule, spanned over 11 days (Table 1).

**Table 1.**
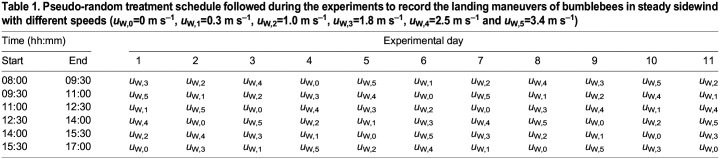
**Pseudo-random treatment schedule followed during the experiments to record the landing maneuvers of bumblebees in steady sidewind with different speeds (*u*_W,0_=0** **m** **s^–1^, *u*_W,1_=0.3** **m** **s^–1^, *u*_W,2_=1.0** **m** **s^–1^, *u*_W,3_=1.8** **m** **s^–1^, *u*_W,4_=2.5** **m** **s^–1^ and *u*_W,5_=3.4** **m** **s^–1^)**

We used a customized machine-vision-based videography system (Straw et al., 2011) to track in real-time (at 175 Hz) all three-dimensional flight movements in the wind tunnel test section (Fig. 2A,B). The video system consisted of four high-speed cameras with a custom-built array of infrared LED panels for illumination (for details, see Goyal et al., 2021). Based on the position coordinates of the tracked bumblebees, we reconstructed (and stored) the 3D flight trajectories of each bumblebee in a global Cartesian coordinate system, which was attached to the center of the specific landing surface, with the *y*-axis pointing normal to the landing surface into the tunnel, the *z*-axis vertically upward and the *x*-axis in the downstream direction of the air flow (Fig. 2C). Thus, different coordinate systems were defined at the hive side and at the food source. The coordinate system at the hive side is a right-handed system, whereas the system at the food source is left-handed. This way, the wind always moved in the positive *x*-direction (Fig. 2C). Note that this method allows for tracking individual bumblebees, but it did not allow us to link these tracks to specific individuals within the hive.

### Estimation of state variables

We extracted all flight trajectories in which bumblebees landed on one of the landing platforms, using a previously designed selection procedure (Goyal et al., 2021). These tracks were divided into landings on the landing platform at the food source or at the hive side. Additionally, we characterized the landing type of each track, being either a landing from free-flight or directly after taking off (from the ground or from the landing platform on the opposite side).

We filtered all tracks using a low-pass second-order two-directional Butterworth filter (cut-off frequency=20 Hz, filtfilt in MATLAB 2020a) and stored the filtered track as space–time arrays **X**=(*x*,*y*,*z*,*t*), with time *t* set to zero at the end of the landing maneuver (i.e. when a bumblebee was closest to the landing surface). We used a second-order central differentiation scheme to compute the corresponding velocity and acceleration arrays of the flying bumblebee. Both were defined in the landing-platform coordinate system as the ground velocity **U**_G_=(*u*_G_,*v*_G_,*w*_G_) and body acceleration vector **A**=(*a_x_*,*a_y_*,*a_z_*), respectively (Fig. 2D).

In addition to the ground velocity of the bumblebee **U**_G_, we also recorded at each time-step the wind velocity **U**_W_=(*u*_W_,0,0) and the air velocity of the bumblebee defined as **U**_A_=**U**_G_–**U**_W_=(*u*_A_,*v*_A_,*w*_A_)=(*u*_G_–*u*_W_,*v*_G_,*w*_G_). Here, *u*_W_ is the wind velocity in the landing-platform coordinate system, and wind speed *U*_W_ is the magnitude of the wind velocity vector. Vector **U**_A_ is the relative air velocity experienced by the bumblebee, ignoring the effect of the bumblebee itself on the airflow. Thus, **U**_A_ depends on both the wind velocity and the ground velocity of the bee, and therefore it changes with time. The magnitude of **U**_A_ is denoted as airspeed *U*_A_.

To describe the approach of bumblebees towards the landing surface, we computed the temporal dynamics of four state variables: approach distance from the surface *y*(*t*), approach speed *V*(*t*)=−*v*_G_(*t*), approach acceleration *A_y_*(*t*)=−*a_y_*(*t*), and the relative rate of optical expansion that a bumblebee experiences owing to its motion normal to the landing surface *r*(*t*)=*V*(*t*)/*y*(*t*) (Fig. 2D–F). We used the velocity perpendicular to the surface for the computation of relative rate of expansion, as bumblebees landing in still air have been shown to progressively increase and decrease this component as they advance towards the landing surface (Goyal et al., 2021).

We describe the vertical flight kinematics and those in the wind direction, parallel to the landing platform, using six additional state variables: (1,2) streamwise and vertical position relative to the landing platform [*x*(*t*) and *z*(*t*), respectively], (3,4) streamwise and vertical velocity components [*U*(*t*)=*u*_G_(*t*) and *W*(*t*)=*w*_G_(*t*), respectively], and (5,6) streamwise and vertical acceleration [*A_x_*(*t*)=*a_x_*(*t*) and *A_z_*(*t*)=*a_z_*(*t*), respectively]. Here, positive *x*-values indicate downwind position, velocity and acceleration; positive *z*-values define vertical upward position, velocity and acceleration (Figs 2C). See List of symbols for all symbol definitions.

### Extraction and characterization of constant-*r* segments and entry segments

To determine whether bumblebees in the presence of winds use a similar modular landing strategy as in still air (Goyal et al., 2021), we applied the same analysis approach as used in that study. The algorithm developed for this identifies track segments in which a bumblebee kept the relative rate of expansion nearly constant (called constant-*r* segments). The corresponding response is called the ‘steady-state’ flight response. We characterize these constant-*r* segments with the average values of the state variables (X*={x*,y*,z*}, U*={U*,V*,W*}, A*={A_x_^*^,A_y_^*^,A_z_^*^}, r*), where r* is referred to as a set-point of relative rate of expansion (Fig. 2E). It is an estimate of the *r*-value that a landing bumblebee aims to fly at using its sensorimotor control system (Goyal et al., 2021).

The set-point extraction algorithm used to identify the constant-*r* segments depends on the sensitivity factor *f*, which restricts the variation allowed around the mean r* for a segment to be identified as a constant-*r* segment. The sensitivity factor *f* characterizes the number of standard deviations σ around the mean r* that are included in the set of constant-*r* segments. This algorithm uses generalized *t*-distributions. The sensitivity factor is multiplied by a scale parameter σ of these distributions to obtain the plausible intervals of variables that determine the constancy of *r* in a track segment (Goyal et al., 2021). Here, we present the results for sensitivity factor *f*=1, but our results remain similar for a wide range of sensitivity factors (0.25≤*f*≤2.5), albeit with variable numbers of identified constant-*r* segments.

To analyze the sensorimotor control response of bumblebees in different wind conditions, we used a second previously developed algorithm (Goyal et al., 2022). This algorithm identifies the track segments that precede the constant-*r* segments and contain a monotonic variation (increase or decrease) of relative rate of expansion (Fig. 2E). In still air, this monotonic variation of *r* is the transient response of the sensorimotor control system when converging to the optic expansion rate set-point (Goyal et al., 2022). We refer to these segments as entry segments, and the corresponding flight responses as transient responses. We characterize each entry segment with six state variables (Fig. 2G): (1) optical expansion acceleration 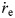, (2) mean body acceleration 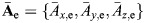, (3) the required step-change in relative rate of expansion Δ*r*_e_, (4) the associated set-point r*, (5) the initial position at the start of the entry segment **X**_0_={*x*_0_, *y*_0_, *z*_0_} and (6) the mean air velocity during the entry segment 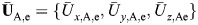.

Here, we use 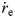 as a performance measure of the transient sensorimotor control response, as it dictates how fast a bumblebee reaches the expansion rate set-point r*. For each entry segment, it is estimated from a linear regression: *r*(*t*) =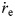
*t*+*c*+ε, where *c* and ε denote the intercept and residuals, respectively. The coefficient of determination *R*^2^ for this regression was very high (0.980 [0.96 0.99], median [interquartile range]). Moreover, the difference between the actual flight distance covered and the analytically computed flight distance if the bumblebees had performed the motion exactly at the estimated expansion acceleration within the identified entry segments was also very low (0.0011 m [–0.0015 m, 0.0042 m], median [interquartile range]). Thus, this linear regression captured well the motion during entry segments in all tested wind conditions (Fig. 2G). Mean body acceleration 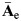 was computed as a ratio of change in ground velocity and travel time during an entry segment. For 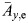
>0, the bumblebee accelerates towards the landing surface; for 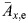
>0, the bumblebee accelerates downwind; for 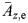
>0, the bumblebee accelerates upwards.

Our algorithms for extracting the constant-*r* segments and entry segments do not capture all the set-points or entry phases that bumblebees exhibit during landing. For details about the limitations of each algorithm, see Goyal et al. (2021) and Goyal et al. (2022), respectively. We overcome these limitations by using thousands of landing maneuvers to describe the influence of winds on the landing dynamics of bumblebees.

### Characterization of the hovering phases exhibited by landing bumblebees

During landings, bumblebees may also rapidly break, causing them to greatly reduce their approach flight speed, and sometimes even hover or fly away from the landing surface (Fig. 2E). These low ground-velocity flight phases are commonly described as hover phases (de Vries et al., 2020; Goyal et al., 2022; Reber et al., 2016), and we use this terminology here. Hover phases are potentially unfavorable as they tend to increase landing duration, which is energetically costly (Reinhold, 1999), in particular in the presence of winds (Shepard et al., 2016). Moreover, for foraging bees, an increase in landing time can reduce their floral visitation rate, and hence their energy gain (Hansen et al., 2002; Roubik, 1978).

To characterize how often bumblebees landing in a sidewind exhibited such hover phases, and how this affected landing duration, we identified these hover phases within all recorded landing maneuvers. We did so by defining hover phases as sections during which the bumblebee reduced its approach flight speed *V* to below 0.05 m s^−1^. We then divided each landing trajectory into sections at four distances from the landing platform (0.05<*y*_1_<0.10 m, 0.10<*y*_2_<0.15 m, 0.15<*y*_3_<0.20 m and 0.20<*y*_4_<0.25 m), and recorded the number of hover phases exhibited in each section. We did this for all landing maneuvers that started beyond section four (*y*=0.25 m). We applied a generalized linear mixed-effects model to the resulting hover phase distributions, to test how the probability of exhibiting a hover phase *P*_hover_ varied with wind speed, among the four *y*-sections and between the two landing types (landings after take-off and from free-flight). See Statistical analyses below for details.

### Quantification of the landing performance of bumblebees

To assess the overall landing performance of bumblebees at different sidewinds, we computed the travel time Δ*t* for each landing approach, which depicts how long bumblebees remain airborne during landing. The latter directly affects both the energetic cost and time budget of the landing maneuvers of foraging bumblebees. Therefore, minimizing Δ*t* can be a driving factor for maximizing landing performance. For all landing maneuvers that started beyond *y*=0.25 m, we computed Δ*t* as the time that a bumblebee takes when traveling from *y*=0.25 to *y*=0.05 m (distance 0.2 m). We then applied a generalized linear mixed-effects model to the travel time results, to test how Δ*t* varied with wind speed, and between two landing types (landings after take-off and from free-flight). See Statistical analyses below for details.

### Statistical analyses

We used R 4.0.3 (https://www.r-project.org/) for statistical analyses. We developed linear mixed-effects models and a generalized linear mixed-effects model (using the R functions lmer and glmer, respectively). Wherever relevant, we used the approach sequence, the landing side (whether a bumblebee landed on the side of the hive or food source), the day of the experiment and the timeslot during the day as random intercepts. Probability values *P*<0.05 were considered statistically significant. For *post hoc* comparisons, we used Bonferroni correction (using the emmeans package in R) to adjust the statistical significance values. We reported results and statistical estimates as mean values with either 95% confidence intervals (CI), ±s.e.m. or ±s.d., depending on what is most appropriate.

The statistical models for testing how sideways wind affects the landing kinematics of flying bumblebees are divided into three types: (1) we tested how the frequency of landing on the platform is affected by wind speed; (2) we tested how the average landing kinematics is affected by wind speed; and finally (3) we assessed in a series of tests how individual bumblebees changed their landing behavior in response to side wind. The separate tests are described below in detail.

#### Testing how landing frequency depends on wind speed

To test how winds influenced the landing frequency of bumblebees, we used two linear mixed models to find how the average number of landing approaches per hour (*N*) varied with the wind conditions. The two models correspond to two landing types (landing from a free-flight or directly after a take-off) and had the time of day and the day of the experiment as random factors. The statistical model developed is expressed as:
(1)

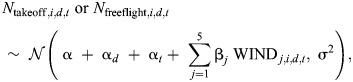
where *N*_takeoff,_*_i_*_,_*_d_*_,_*_t_* and *N*_freeflight,_*_i_*_,_*_d_*_,_*_t_* are the number of landing maneuvers per hour for landing after take-off and from free-flight, respectively, for the *i*th measurement from the *d*th day and *t*th time-slot; α is the regression intercept for zero wind speed (overall intercept); α*_d_* is the day-specific intercept; α*_t_* is the time-slot-specific intercept; WIND*_j_* indicates *j*th wind in the set {0*.*3,1*.*0,1*.*8,2*.*5,3*.*4 m s^−1^}; WIND*_j_*_,_*_i_*_,_*_d_*_,_*_t_* indicates whether the *j*th wind condition is present for the *i*th measurement on the *d*th day and *t*th time-slot (0=no, 1=yes); β*_j_*∀*j*∈{1,2,*…*,5} represents the differences of fixed-effects (wind conditions) from the overall intercept; and σ is the residual standard deviation. The statistical output and the results from *post hoc* tests are given in Table S1.

#### Modeling how the landing kinematics of the average bumblebee depends on wind speed

We used a linear mixed model to test how side wind affects the average landing maneuvers of bumblebees. The dependent parameter is the mean relative rate of expansion during the approach flight (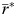), fixed factors are wind speed, landing type and their interactions, and random factors are day of the experiment, landing approach number and landing side (whether landing disc is located on the hive side or the food source side). The model is defined as:
(2)

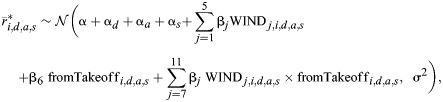


where 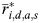 is the relative rate of expansion for the *i*th measurement from the *d*th day (*d*∈{1,2,*…*,11}), *a*th measured landing approach (*a*∈{1,2,…,19,421}) and landing side *s* (*s*=1 for hive side and *s*=2 for food-source side); α is the regression intercept for zero wind speed and landing from free-flight (overall intercept); *α_d_* is the day-specific intercept; α*_a_* is the landing-approach-specific intercept; α*_s_* is the landing-side-specific intercept; WIND*_j_* indicates *j*th wind in the set {0*.*3,1.0,1.8,2.5,3*.*4 m s^−1^}; WIND*_j_*_,_*_i_*_,_*_d_*_,_*_a_*_,_*_s_* and fromTakeoff*_i_*_,_*_d_*_,_*_a_*_,_*_s_* indicate whether the *j*th wind speed and take-off are present for the *i*th measurement from the *d*th day, *a*th landing approach and landing side *s* (0=no, 1=yes); β*_i_*∀*i*∈{1,2,…,11} represents the differences of the fixed-effects and interaction terms from the overall intercept; and σ is the residual standard deviation. The statistical output, along with *post hoc* tests, is given in Table S2.

#### Modeling how wind speed affects the landing maneuvers of individual bumblebees

We used several statistical models to test how individual bumblebees change their landing kinematics in response to a side wind.

First, we used a linear mixed model to test how bumblebees adjusted their optic expansion rate set-point (r*) in response to side winds. We first constructed a full model with log(r*) as a response variable, fixed factors were log(y*), wind speed, landing types and their interactions, and random factors were day of the experiment, landing approach and landing side. Model dredging revealed that only the interaction term log(y*)×landingType was significant; therefore, the resulting reduced model was defined as:
(3)

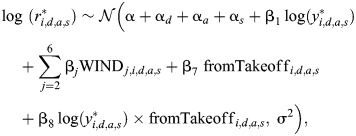
where *r*_i,d,a,s_^*^ and *y*_i,d,a,s_^*^ are set-points of relative rate of expansion and mean distance, respectively. The definition of other parameters are the same as for the average landing maneuver model (Eqn 2). The statistical output, along with *post hoc* tests, is given in Table S3.

Second, we used two linear mixed-effects models to test how sidewind affects the optical expansion-accelerations during transient response phase (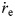), and the corresponding body acceleration towards the landing platform (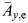). In both models, fixed factors were wind speed, the starting distance from the landing surface (*y*_0_), the required step-change in relative rate of expansion (Δ*r*_e_), the final set-point to reach (r*) and the starting condition of the landing maneuver (whether the landing is from a free-flight or after a take-off). Random intercepts were the day of the experiment, the landing approach and the landing side (whether the landing disc is located on the hive side or the food-source side).

We used model dredging to construct the reduced models, which revealed that for both models, only the interaction terms log(Δ*r*_e_)×log(*y*_0_) and log(Δ*r*_e_)×log(r*) were significant; therefore, the resultant reduced models are defined as:
(4)

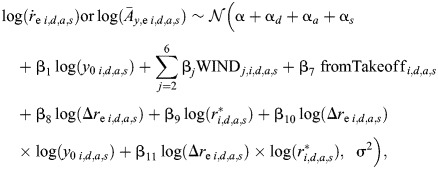


where the parameter definitions are the same as for the average landing maneuver model (Eqn 2). The statistical outputs of the 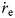 and 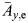 models are given in Table S4.

Note that the reduced models in still air are similar to the ones identified previously (Goyal et al., 2022), except the log(Δ*r*_e_)×log(r*) term. This term is statistically significant in the present study owing to the larger dataset of landing maneuvers being used here as compared with the previous study. Despite its statistical significance, this interaction term had little effect on the response variables (Table S4).

Third, we used two additional linear mixed-effects models to test how sidewind affects the mean sideways and upward body accelerations during the transient response phase (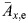 and 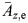, respectively). For both models, the fixed factors were wind speed, landing type (landing from a free-flight or after a take-off) and the respective starting position for that specific acceleration (*x*_0_ and *z*_0_ for the 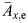 and 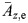 models, respectively). In both models, we used the day of the experiment, the landing approach and the landing side as random intercepts.

Model dredging revealed that for the 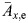 model all interactions were significant, and thus the final model is defined as:
(5)

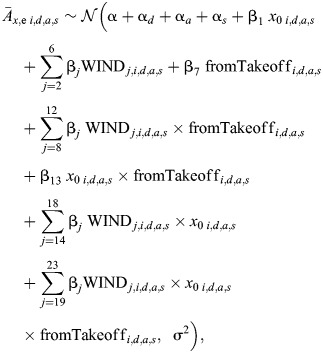


where parameter definitions are the same as for the average landing maneuver model (Eqn 2). The statistical output of 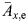 model is given in Table S4.

For the 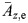 model, model dredging and subsequent *post hoc* tests revealed that the vertical accelerations did not significantly vary with wind speed, and thus we excluded this model from any further analyses.

Fourth, we used a generalized linear mixed model to test how the probability of exhibiting a hover phase during the approach flight phase (*P*_hover_) varied with wind speed, landing type (landing from free-flight or take-off) and distance to the surface *y* (divided into four regions: 0*.*05*<y*_1_≤0*.*10 m*,* 0*.*10*<y*_2_≤0*.*15 m*,* 0*.*15*<y*_3_≤0*.*20 m and 0*.*20*<y*_4_≤0*.*25 m), and their interactions. Random factors are day of the experiment, the landing approach and the landing side. Model dredging revealed that all two-way interactions between these explanatory variables were significant. Therefore, the final reduced model is defined as:
(6)

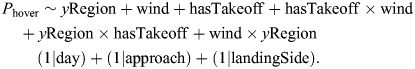


The statistical output of the *P*_hover_ model is given in Table S5.

Fifth, we used a linear mixed model to test how the travel time of bumblebees Δ*t* varied with wind speed and the landing type (landing from free-flight or take-off). In this model, day of the experiment and landing side (whether landing disc is located on the hive side or the food source side) are used as random factors. The model dredging revealed that two-way interaction between the explanatory variables were significant. Therefore, the final reduced model is defined as:
(7)

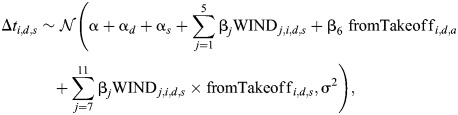
where parameter definitions are the same as for the average landing maneuver model (Eqn 2). The statistical output is given in Table S6.

## RESULTS

We tracked the three-dimensional flight kinematics of 19,421 landing approaches of bumblebees in the five sidewind speeds, and a zero-wind control case (*U*_W_=0 to 3.4 m s^−1^) (Table 1; Database S1 in Mendeley Data, https://doi.org/10.17632/mww9m8r3dk.1). Among these, 16,374 tracks represented landings from free-flight, and 3047 landings were performed directly after take-off from the opposite landing platform or the ground. These landings resemble those when bumblebees move between flower patches and the hive, or when visiting multiple flowers within a single flower patch, respectively.

### Bumblebees land less often in high sidewind

**Fig. 3.**
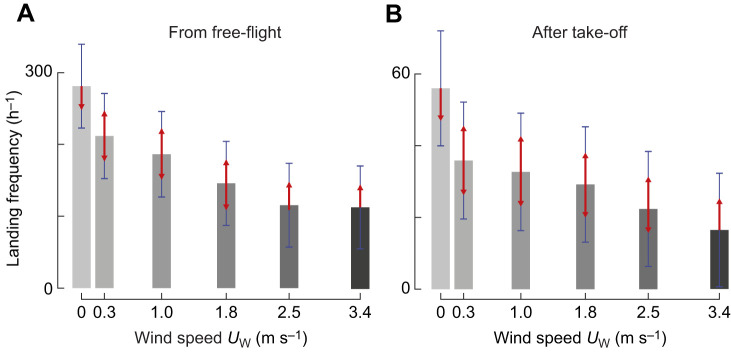
**Bumblebees perform fewer landings at higher windspeeds.** (A,B) Landing frequency, as landings per hour in different sidewind velocities for landings from free flight (A) or directly after take-off (B). Grey bars show the average landing frequency, blue bars show 95% confidence intervals, and non-overlapping red arrows indicate statistically significant differences between conditions (Eqn 1; Table S1).

Landings from free-flight occurred 60% less in the highest winds (*U*_W_=3.4 m s^−1^) than in still air (*N*=112.2±27.8 h^−1^ and *N*=280.8±28.3 h^−1^, respectively; mean±s.e.m.; Fig. 3A; Table S1). Landings after take-off occurred 70% less often in the highest wind condition (*U*_W_=3.4 m s^−1^) than in still air (*N*=16.5±7.7 h^−1^ and *N*=56.0±7.8 h^−1^, respectively; Fig. 3B, Table S1). Thus, sidewinds reduce the landing frequency of foraging bumblebees.

### The average landing approach in different sidewind speeds

**Fig. 4.**
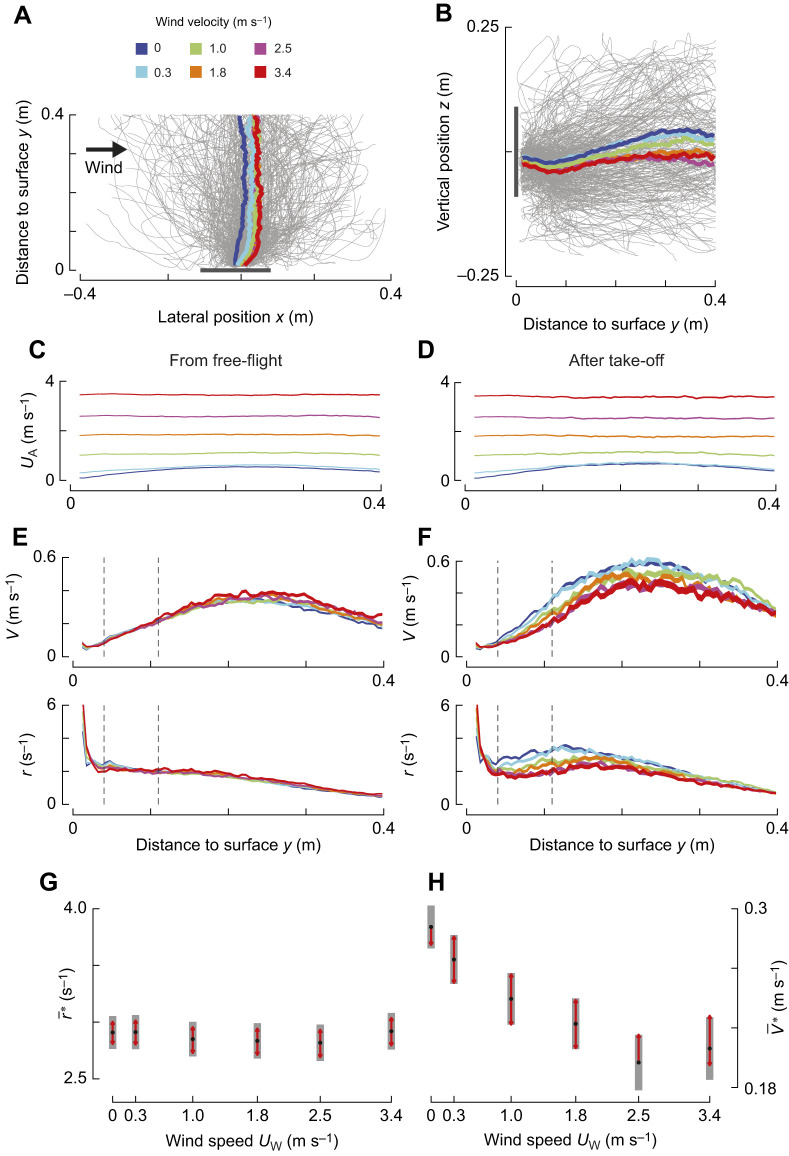
**Effect of sidewind on the average approach kinematics of landing bumblebees.** (A,B) Top and side views of landing maneuvers of bumblebees in all tested wind conditions. Grey lines show every 70th recorded landing maneuver (*n*=281 tracks). Colored lines show mean landing maneuvers in different wind conditions (key in A). (C–H) Differences in landing kinematics with wind speed (color-coded as in A); left and right panels show results for landings from free-flight and after take-off, respectively. The kinematic parameters are airspeed *U*_A_ (C,D), approach speed *V* and optic expansion rate *r* (E,F), averaged over the analyzed landings; line thickness indicates the standard error of the means. (G,H) Linear mixed model (Eqn 2) estimates of the average relative rate of expansion set-point 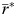 and resulting average approach velocity 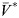 at *y*=0.075 m, for landings from free-flight (G) and after take-off (H), respectively (Table S2). Black dots depict estimated means, grey bars show 95% confidence intervals, and non-overlapping red arrows indicate significant differences between conditions. Two vertical dashed grey lines in E and F show the distance range (0.04<*y*<0.11 m) for modeling 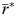.

In all tested sidewinds, bumblebees flew on average approximately perpendicular to the landing surface (Fig. 4A,B). Bumblebees experienced higher airspeeds *U*_A_ in higher sidewinds (Fig. 4C,D). Thus, they had to generate higher compensatory sideways forces and torques during their landing approach. Most importantly, they needed to compensate for the additional drag force in the wind direction. On average, they did this in all tested wind conditions, though with a slight lateral drift in the wind direction and a small height loss (Fig. 4A,B).

On average, bumblebees first gradually increased and then decreased their approach velocity *V* as they approached the landing surface (Fig. 4E,F). Landings from take-off showed, on average, a higher maximum approach velocity *V* than those from free-flight, in particular for the lowest wind speeds. During their deceleration phase from free-flight (0.04<*y*<0.11 m), the bumblebees flew on average at a nearly constant average set-point of optical expansion rate 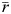
* for all applied wind speeds. In comparison, landings from take-off showed a slight reduction in 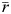
* for most wind speeds (0–1.8 m s^−1^).

During landing from free-flight, bumblebees had similar average set-points in all wind conditions (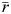
*=2.89±0.08 s^−1^ at *U*_W_=0 m s^−1^ and 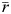
*=2.90±0.08 s^−1^ at *U*_W_=3.4 m s^−1^; Fig. 4E,G; Table S2), and thus similar approach velocities throughout the deceleration phase. In contrast, when bumblebees landed shortly after a take-off, they decreased their average set-point with increasing wind speed (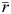
*=3.84±0.10 s^−1^ at *U*_W_=0 m s^−1^ and 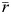
*=2.75±0.14 s^−1^ at *U*_W_=3.4 m s^−1^; Fig. 4F,H; Table S2). Hence, only at low wind speeds (*U*_W_<1.7 m s^−1^) did landings from take-off have higher set-points than landings from free-flight; at high sidewinds, the set-points were similar (Fig. 4G,H).

### At all windspeeds, landing bumblebees stepwise modulated their optical expansion set-point

**Fig. 5.**
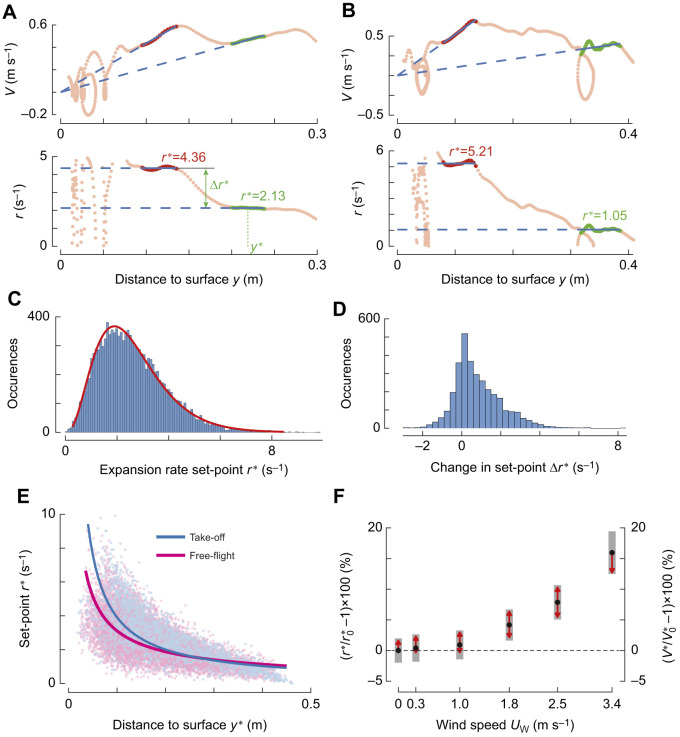
**Bumblebees landing in a sidewind fly at higher optical expansion set-points than those landing without wind.** (A,B) Approach velocity *V* and relative rate of expansion *r* versus distance *y* for bumblebees landing in a 3.4 m s^−1^ sidewind, after a take-off (A) and from free-flight (B). Two constant-*r* segments (green and red, respectively) and their optical expansion rate set-points r* (blue) are highlighted. (C) Histogram of all identified set-points r* (*n*=12,338), with gamma distribution fit in red. (D) Histogram of change Δr* between two consecutive constant-*r* segments in a landing maneuver, as defined in A (*n*=3241). (E) Variation of r* with corresponding distance y* (defined in A), for landings after take-off (blue) and from free-flight (pink). Solid lines depict output of linear mixed models (Eqn 3; Table S3). (F) Effect of wind on r* and V*, defined as percentage change relative to values at zero wind (*r*_0_^*^ and *V*_0_^*^). Black dots depict estimated means, grey bars show 95% confidence intervals, and non-overlapping red arrows indicate significant differences.

The flight paths of individual bumblebees deviate substantially from the average behavior (Fig. 4A,B) (Goyal et al., 2021). Therefore, we also analyzed the individual landing maneuvers (Fig. 5A,B) (Goyal et al., 2021, 2022, 2023). Using a set-point extraction algorithm (Goyal et al., 2021), we identified 12,338 constant-*r* segments in 9097 landing tracks (for sensitivity factor *f*=1) (Fig. 5C); its distribution approximates a gamma distribution (median r*=2.41 s^−1^, *a*=3.74 [3.65–3.83], *b*=0.69 [0.68–0.71], mean [95% CI]) (Evans et al., 2000).

Out of the 9097 landing tracks with constant-*r* segments, 2632 had more than one constant-*r* segment (see Fig. 5A,B for examples). In these tracks, bumblebees switched from one set-point to another 3241 times, which occurred in all wind conditions (Fig. 5D). In 76% of these 3241 set-point transitions, bumblebees switched to a higher set-point, resulting in a set-point increase of Δr*=1.24±1.09 s^−1^, mean±s.d.). For the remaining 24% of the transitions, bumblebees reduced their set-point with Δr*=−0.48±0.48 s^−1^.

Bumblebees increased their optic expansion set-point with decreasing distance to the surface, whereby a linear relationship occurred between the logarithmic transformations of r* and the corresponding mean distance to the surface y* (Fig. 5E; Table S3). Our linear mixed-effects model (Eqn 3) showed that the slope of the linear regression between these logarithmic transformations (*m*) was significantly different between landing from free-flight and landings after take-off (*m*=−0.727*±*0.008 and −0.960±0.017, respectively). Surprisingly, these dynamics were independent of wind speed (Table S3).

### With increasing windspeeds, bumblebees approach the landing surface at higher optic expansion set-points

Although *m* did not vary significantly with wind speed, the baseline optic expansion set-points at which bumblebees land in a sidewind were higher than for landings in still air (Fig. 5F). This increase in set-point with wind occurred at all distances to the surface, for landing from both free-flight and take-off (Fig. 5F, Table S3). At sidewinds of 2.5 and 3.4 m s^−1^, bumblebees flew on average at an 8% and 16% higher set-point than in still air, respectively. Hence, bumblebees exhibited higher set-points in faster winds, and thereby flew faster towards the surface in higher windspeeds.

### Bumblebees exhibit faster sensorimotor control responses in higher windspeeds

**Fig. 6.**
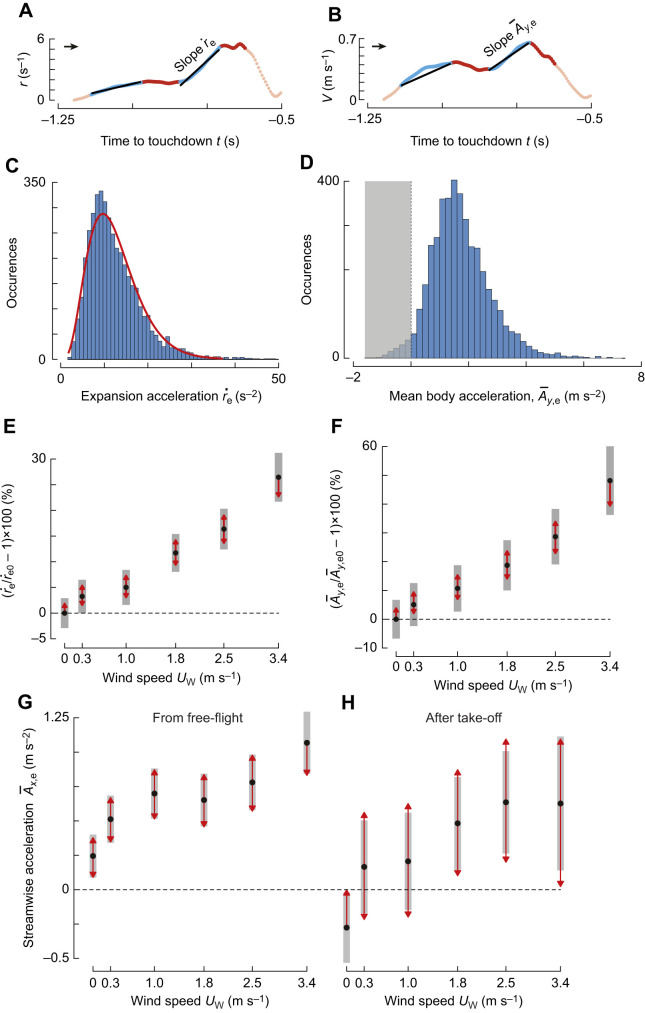
**In sidewind, bumblebees accelerate faster towards the landing platform as well as along the sidewind direction during the entry segments.** (A,C,E) Effect of sidewind speed on expansion acceleration 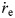 during the entry segments (Eqn 4; Table S4). (B,D,F) Effect of sidewind speed on mean accelerations towards the landing platform 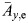 during entry segments (Eqn 4; Table S4). (A,B) Temporal dynamics of optic expansion rate *r* (A) and approach velocity *V* (B), including acceleration parameter definitions. Time is defined relative to touchdown (*t*=0 s), and black arrows indicate direction of time. (C,D) Histograms of occurrences of 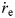 (C) and 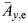 (D), for all identified entry segments with increasing optical expansion rate (*n*=4221). (C) Red line shows the fitted gamma distribution. (D) Bumblebees accelerated 94% of the times towards the landing surface (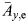
>0) (E,F) Effect of wind on 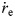 (E) and 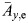 (F), defined as percentage change relative to values at zero wind (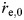 and 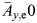). (G,H) Effect of sidewind on sideways mean body acceleration during the entry segments 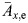, for landings from free-flight (G) and after take-off (H) (Eqn 5; Table S4). This effect is shown for 50th percentile lateral position of bumblebees at the start of the entry segments, *x*_0_= 0.03 m; similar effect is observed for the 25th and 75th percentiles, albeit with slightly different values (Table S4). Black dots depict estimated means, grey bars show 95% confidence intervals, and non-overlapping red arrows indicate significant differences.

The stepwise modulation of the set-point of optical expansion rate results in entry track segments that precede these constant-*r* segments, and contain the transient response of the sensorimotor control system (Fig. 2E,G). Using an entry-segment extraction algorithm (Goyal et al., 2022), we linked 4374 constant-*r* segments with a respective entry segment (Fig. 6). Examples of these entry segments are shown in Figs 1B, 2E,G and 6A,B.

In 97% of these entry segments, bumblebees increased their optical expansion rate, and in only 3% did they decrease it (Fig. 6D). We focused further analyses on the 97% entry segments with increasing optical expansion rate, which was approximated by a gamma distribution (median 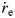
=11.07 s^−2^, *a*=4.55 [4.4–4.7], *b*=2.7 [2.6–2.9]) (Fig. 6C) (Evans et al., 2000).

Bumblebees increased the optical expansion acceleration with sidewind speed, and thus landing bumblebees reached their set-points faster at higher windspeeds (Fig. 6E). For example, in a 2.5 and 3.4 m s^−1^ sidewind, bumblebees reached their set-point 16% and 27% faster than in still air, respectively (Fig. 6E, Table S4). This wind effect was observed independently of all covariates (*y*_0_, Δ*r*_e_, r* and landing type). Hence, the transient response of the sensorimotor control system of landing bumblebees increased with higher sidewinds, allowing them to reach their set-point faster.

### During entry segments, bumblebees accelerate faster towards the landing surface in higher windspeeds

Landing bumblebees use transient responses to accelerate towards the landing surface (Goyal et al., 2021, 2022). To study the effect of sidewind on these transient responses, we computed the mean body acceleration towards the landing surface (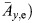 in all 4374 identified entry segments (see examples in Figs 2G and 6B).

In 153 of these 4374 entry segments, bumblebees reduced their approach speed (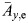
<0) to decrease their optical expansion rate (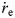
<0). Among the remaining 4221 entry segments, bumblebees accelerated on average towards the landing surface (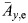
>0) in 4102 entry segments and weakly decelerated (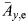
<0) in 119 entry segments to increase their optical expansion rate (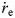
>0) (Fig. 6D). Hence, in 94% of all identified entry segments, bumblebees landing in a sidewind used the transient response of their sensorimotor control system to accelerate towards the landing surface. Moreover, the body accelerations produced by the bumblebees during these entry segments correspond mostly to an increase in relative rate of optic expansion.

Using a linear mixed model (Eqn 4), we found that bumblebees accelerated faster towards the landing surface in higher sidewind speeds (Fig. 6F; Table S4). For example, in a 2.5 and 3.4 m s^−1^ sidewind, bumblebees accelerated on average 29% and 48% faster towards the landing platform than in still air, respectively. This behavior was independent of all covariates (*y*_0_, Δ*r*_e_, r* and landing type; Table S4).

### While accelerating towards the landing surface, bumblebees also accelerate in the sideways wind direction

To land accurately on a vertical surface, flying bumblebees need to control not only their approach speed, but also their position and speed parallel to the platform. Flying insects control this using aerodynamic force-induced accelerations, and thus we here assessed how sideways accelerations *A_x_* are affected by sidewinds.

On average, our bumblebees flew towards the landing platform in a curved flight path that was nearly perpendicular to the platform (Fig. 4A). Therefore, the net acceleration parallel to the platform throughout the complete path should be close to zero. In contrast, for bumblebees landing in a sidewind, the sideways accelerations during the transient response phases (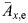) were significantly different from zero (Fig. 6G; Table S4).

Our linear mixed model analysis (Eqn 5) shows that the mean sideways accelerations during the transient phases increase approximately linearly with sidewind speed (Fig. 6G; Table S4). As a result, for landings from free-flight, the average sideways acceleration during the transient phase increases from 0.25 m s^−2^ at the lowest windspeed to 1.09 m s^−2^ in the highest sidewind speed (3.4 m s^−1^). For landings after take-off, the variations in sideways acceleration per condition are higher, and maximum accelerations lower (Fig. 6G; Table S4).

These positive sideways accelerations (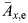
>0) indicate an acceleration in the wind direction, and so bumblebees start to drift downwind during these transient phases in a sidewind. To compensate for these downwind drifting motions, the landing bumblebees would need to produce upwind control forces during other phases of their approach flight.

### Bumblebees hover more often in higher windspeeds and closer to the landing surface

**Fig. 7.**
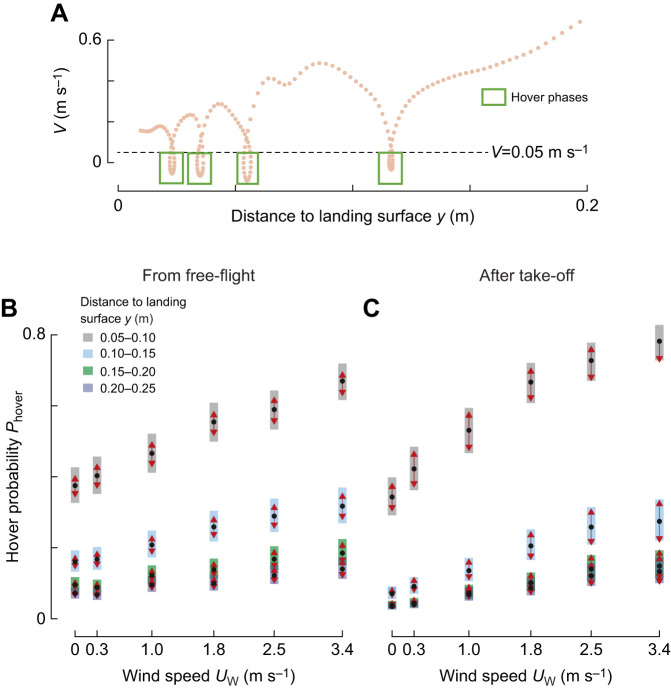
**Bumblebees generate more hover phases when flying in higher sidewinds.** (A) Approach velocity *V* versus distance *y*, with each hover phase (*V*<0.05 m s^−1^) highlighted with a green box. (B,C) Estimated probabilities that a bumblebee transitions to a hover phase at different wind speeds (abscissa), and different distances *y* (color-coded), for landings from free-flight (B) or after take-off (C) (Eqn 6; Table S5). Black dots depict estimated means, grey bars show 95% confidence intervals. Non-overlapping red arrows indicate significant differences.

Bumblebees approaching a landing surface occasionally exhibit moments of near-zero or negative approach velocities (Fig. 7A). Here, we identified these so-called hover phases as track segments in which the approach speed dropped below 0.05 m s^−1^ (Fig. 7A). Using a generalized linear mixed model (Eqn 6), we show that bumblebees exhibited more hover phases in faster winds, for both landing from free-flight and from take-off, and at all distances from the surface (Fig. 7B,C; Table S5). Moreover, all explanatory variables had statistically significant two-way interactions.

#### The effect of distance and landing type on hover probability

For both landing types, hovering probability increased with decreasing distance to the platform; this distance effect on hovering probability was 65% larger in landing from take-off than in those from free-flight (Fig. 7B,C). For landings initiated directly after take-off, hover probability was more than eight times higher closest to the platform (0.05<*y*_1_<0.10 m) than at the furthest analyzed distance (*P*_hover_=0.59±0.03 for 0.05<*y*_1_<0.10 m and *P*_hover_=0.07±0.01 for 0.20<*y*_4_<0.25 m, results are averaged over windspeeds). For landing from free-flight, the equivalent hover probabilities differed only by a factor of five (*P*_hover_=0.51±0.03 for 0.05<*y*_1_<0.10 m, and *P*_hover_=0.10±0.01 for 0.20<*y*_4_<0.25 m).

#### The effect of distance and windspeed on hover probability

At all distances from the landing platform, hovering probability increases with sidewind speed (Fig. 7B,C; Table S5). This effect of sidewind on hovering probability depends significantly on the distance from the landing platform. For example, at the furthest tested distance (0.20<*y*_4_<0.25 m), hover probability in the fastest tested windspeed was 2.8 times that in still air (*P*_hover_=0.14±0.02 at *U*_W_=3.4 m s^−1^, and *P*_hover_=0.05±0.01 at *U*_W_=0 m s^−1^, results are averaged over landing types). At the closest tested distance (0.05<*y*_1_<0.10 m), hover probability increased only a factor two from no wind to highest windspeed (*P*_hover_=0.73±0.02 at *U*_W_=3.4 m s^−1^, and *P*_hover_=0.36±0.02 at *U*_W_=0 m s^−1^).

#### The effect of landing type and windspeed on hover probability

Finally, hover probability consistently increases with sidewind speed, for landings from both free-flight and from take-off (Fig. 7B,C; Table S5). Hereby, wind affected hovering probability more for landings directly after take-off than for landings from free-flight. After take-off, the hover probability in the fastest tested windspeed was 3.8 times the probability in still air (*P*_hover_=0.30±0.03 at *U*_W_=3.4 m s^−1^, and *P*_hover_=0.08±0.01 at *U*_W_=0 m s^−1^, results are averaged over all landing distances). In contrast, after free-flight, the equivalent hover probability ratio was only two (*P*_hover_=0.30±0.02 at *U*_W_=3.4 m s^−1^, and *P*_hover_=0.15±0.01 at *U*_W_=0 m s^−1^). Hence, for landings after take-off, the effect of wind on hovering probability is on average 88% larger than for landings from free-flight.

These combined results show that landing bumblebees generated more hover phases when they encountered higher sidewind speeds, particularly so directly after take-off (Fig. 7).

### For landings from free-flight, travel time does not vary with sidewind speed

The increasing hover prevalence at higher wind velocities might affect the travel time Δ*t* during the landing approaches of bumblebees. We tested this by calculating for each landing approach the time it took the bumblebee to travel from a distance of 0.25 m from the landing platform to a 0.05 m distance. These are the bounds of the four distance bins *y*_1_ to *y*_4_.

**Fig. 8.**
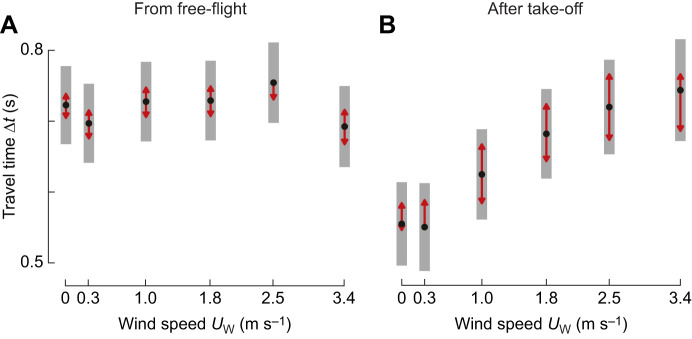
**Travel time of bumblebees landing from free-flight does not vary with windspeed, whereas for landings after take-off, travel time increases with sidewind speed.** (A,B) Effect of sidewind speed on travel time Δ*t*, defined as the time needed to travel from *y*=0.25 m to *y*=0.05 m, for landings from free-flight (A) or after take-off (B) (Eqn 7; Table S6). Black dots depict estimated means, grey bars show 95% confidence intervals. Non-overlapping red arrows indicate significant differences.

Using a linear mixed model (Eqn 7, Fig. 8; Table S6), we show that for landings from free-flight, travel time did not differ significantly between windspeeds, including landings in the highest wind speed and in still air (Δ*t*=0.69±0.03 s at *U*_W_=3.4 m s^−1^, and Δ*t*=0.72±0.03 s at *U*_W_=0 m s^−1^). For landings after a take-off, the travel time was 35% higher in the strongest sidewind as compared with those in still air (Δ*t*=0.74±0.04 s at *U*_W_=3.4 m s^−1^, and Δ*t*=0.55±0.03 s at *U*_W_=0 m s^−1^). Thus, sidewinds negatively affect the landing time directly after take-off, whereas bumblebees landing from free-flight can fully compensate for the detrimental effects of sidewinds on travel time.

## DISCUSSION

Winds are a ubiquitous characteristic of the natural environment of foraging bumblebees (Crall et al., 2017). Here, we studied how bumblebees perform landing maneuvers in steady sidewinds by recording 19,421 landing approaches of bumblebees towards a vertical surface in six different sidewind levels (0 to 3.4 m s^−1^), corresponding to conditions in nature (Crall et al., 2017; Riley et al., 1999).

In all wind conditions, bumblebees made controlled landings by keeping the optical expansion rate *r* approximately constant for brief periods of time during landing (Fig. 5). We call such periods constant-*r* phases, and the corresponding constant-*r* value is the expansion rate set-point r*. Bumblebees tended to stepwise increase r* during the landing approach. This trend of increasing r* with reducing distance is captured by a linear relationship with average slope *m*=−0.843±0.01 between their logarithmic transformations, independent of sidewind speed (Table S3). The rate *m* resembles the previously observed value for bumblebees landing in quiescent air on different landing platforms and at light intensities ranging from dusk to overcast daylight (Goyal et al., 2021), suggesting that the underlying control mechanism is conserved for a wide range of environmental conditions.

The rate *m* is equivalent to the time-to-contact rate 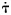
for the landing of birds (Goyal et al., 2021, 2023; Lee et al., 1991), and its magnitude in bumblebees is strikingly similar to that of landing pigeons (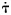
=−0.72) (Lee et al., 1993), hummingbirds (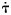
=−0.76) (Lee et al., 1991) and mallards (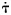
=−0.90) (Whitehead, 2023). Despite the similar slopes for birds and bumblebees, they differ substantially in landing strategy. Birds continuously increase *r* with reducing distance to the surface, whereas bumblebees do it in a stepwise manner.

The stepwise modulation of r* causes landing bumblebees to fly at a range of optical expansion rates while converging towards the new set-point – a typical attribute of a step-response (Ogata, 2010). In still air, these time-evolutions, captured as entry segments, are the transient response of a sensorimotor control system that regulates the optical expansion rate (Goyal et al., 2022). The accelerations (or decelerations) normal to the platform during these entry segments bring the optical expansion rate closer to the desired set-point. Here, we showed that in a sidewind, bumblebees exhibit similar transient flight behaviors when switching between different optic expansion set-points. In addition to the stepwise variation of *r*, bumblebees landing in a sidewind also regularly generated low-velocity flight phases, in which they hover or even briefly fly away from the surface. These hover phases are similar to those observed in landings in still air (de Vries et al., 2020; Goyal et al., 2021; Reber et al., 2016). Next to the similarity between landings in still air and in sidewinds, we also observed important differences in landing kinematics and control, as discussed below.

### Sidewinds increase the optic expansion rate set-points of landing bumblebees

Our analysis showed that landing bumblebees exhibit higher values of r* in faster winds (Fig. 5F), and thus the approach flight speeds during the constant-*r* segments are increased in high sidewinds. This resembles the dynamics found for honeybees landing in a headwind, where the mean set-point of translational optic flow increases with headwind speed (Baird et al., 2021). This suggests that, in addition to the vision-based control system, the airspeed measuring mechanosensory modality also influences the optic expansion rate set-points that landing bumblebees converge to.

### The transient flight response time of landing bumblebees reduces with increasing sidewinds

Here, we showed that bumblebees landing in sidewinds exhibited faster transient responses, expressed as higher optical expansion accelerations (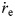) and faster mean body accelerations towards the landing platform (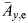). This holds for all covariate values that influence the transient response of bumblebees, including distance from the landing surface (*y*_0_), required step-change in optical expansion rate (Δ*r*) and the associated r*. The increased 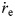 and 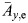 values during the transient response in high sidewind indicate that these bumblebees generate higher thrust forces towards the platform.

Strikingly, the high accelerations towards the platform at higher wind speeds coincide with comparable sideways accelerations in the wind direction (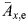). These sideways accelerations are largest in the highest wind speed case, suggesting a wind-induced effect.

When flying in a side wind, a bumblebee would need to produce an upwind-directed aerodynamic control force to prevent accelerating downwind. During the average landing maneuver, bumblebees produce this upwind force as, on average, they tend to land on the middle of the landing platform (Fig. 4). But during the transient response phases of the approach flight this is not the case, as here they accelerate significantly downwind. This suggests that during these phases, sidewind compensation control is compromised. Furthermore, these downwind accelerations cause a detrimental downwind drift, which the animal needs to compensate for during other phases of the landing maneuver.

### Sidewinds increase the hover prevalence in landing maneuvers

We found that the number of hover phases during a landing approach increases with sidewind speed. More hover phases could result in a longer landing duration, which in turn can negatively impact their foraging efficiency (Balfour et al., 2021). Why do bumblebees nevertheless tend to increase the hover phase number in faster winds?

Firstly, next to the negative effect of hovering on landing speed, a hover phase allows the animal to negate the poorly controlled movements that might destabilize the landing maneuver (de Croon, 2016). Therefore, the increase in hovering probability with increasing sidewind might reflect a compensatory response to the reduction in flight control with increasing wind. The most apparent reduction in flight control is observed in the transient response phases of the landing, where in a sidewind, bumblebees accelerate downwind, causing a windward drift in their flight path. A side drift that becomes too large can be negated by performing a hover phase.

Secondly, the increase in the transient response of the sensorimotor control system with wind velocity is analogous to bumblebees operating their visual feedback loop at a higher gain in still air. This increased gain in the *r*-based control loop can result in instabilities in the controller, causing oscillations in the flight path (de Croon, 2016). Oscillations that become too large might trigger a hover response, although other oscillation-controlling mechanisms might also be at play (de Croon, 2016).

Thus, the concomitant increase with wind speed of hover phases and accelerations in transient response phases suggests that bumblebees use the hover phases to reduce the detrimental effects of sidewind on flight control during landing.

### Sidewind increases the travel time of landing maneuvers, but only of landings after take-off

We used travel time of landing approaches as a metric for the landing performance of foraging bumblebees. For landings from free-flight, sidewinds did not affect these times, whereas for landings initiated directly after take-off, travel times increased with sidewind velocity (Fig. 8A and B, respectively).

A second metric for landing performance is the average approach velocity 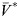 estimated using the average-track-based analysis method (Fig. 4), which shows strikingly similar results as the travel time analysis. The observed 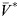 of bumblebees landing from free-flight was constant for all tested wind conditions, whereas for landings directly after take-off, 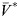 decreased with increasing sidewind speed (Fig. 4G and H, respectively). Hence, the average-track-based analysis method is useful to estimate landing performance metrics such as the average approach velocity. However, it does not capture the observed complex kinematics of bumblebee landing maneuvers.

The combined landing duration and flight speed analyses thus show that bumblebees landing from free-flight can fully compensate for the detrimental effect of sidewind on landing performance, but bumblebees landing directly after take-off cannot. The difference between these cases might be due to the contrasting flight state of these bumblebees. Bumblebees taking off in a sidewind need to first rapidly trim their body and wingbeat kinematics to control for this sidewind, whereas freely flying bumblebees have already fully adapted to this. As a result, landing control directly after take-off might be constraint by this transient wind-compensatory trimming behavior after flight initiation, causing a reduction in landing performance.

These landings performed directly after take-off are most similar to the rapid consecutive landings made by bumblebees when visiting flowers in a single flower patch. Our results contrast with those obtained for honeybees, where an increase in wind speeds did not affect the inter-flower flight duration (Hennessy et al., 2020, 2021). These contrasting results can equally be explained by the above-mentioned hypothetical wind-compensatory trimming mechanism, as honeybees that consecutively visit multiple flowers are also most likely in a free-flying state, where they have fully adapted to flying and landing in windy conditions.

### How bumblebees compensate for the detrimental effects of landing in a sidewind

The primary identified detrimental effect of sidewind on landing of bumblebees are the increased windward accelerations of the bumblebees during the transient response phase. These accelerations cause a downwind side drift that the animal needs to compensate for in order to perform a controlled directed landing on the vertical platform. The most important compensatory mechanism for reducing the side drift is the use of hover phases, as this allows the animal to stop the side drift and realign itself in front of the landing platform. Therefore, we here propose that the increase in hovering prevalence with wind speed allows the animal to reduce the detrimental effect of the windward accelerations during the transient response phases.

Next to this side drift-compensatory mechanism, hovering maneuvers also decrease the landing speed and, consequently, foraging efficiency. Thus, the increased hovering prevalence in high sidewinds can directly explain the observed concomitant increase in landing duration, for landings directly after take-off (Fig. 8B). Despite the negative effects, the animals may have to hover to prevent instabilities in their flight control. In contrast, the duration of landing approaches from free-flight did not differ between wind conditions (Fig. 8A), whereas the hover prevalence did increase with wind speeds (Fig. 7B).

This apparent paradox can be explained by increases of two different aspects of the bumblebee's flight response for landing in a sidewind. First, bumblebees landing in a sidewind fly at higher optic expansion set-points, which leads to higher approach flight speeds (Fig. 5F). Second, bumblebees landing in a sidewind performed more rapid transient flight responses, leading to faster approach accelerations (Fig. 6E,F). These two mechanisms combined tend to reduce the landing duration with increasing sidewind, for both free-flight landings and landings from take-off. For free-flight landings, this compensates approximately for the increased number of hovering bouts (Fig. 8A). For landings from take-off, the increased flight response compensates only for a fraction of the time spent during hovering (Fig. 8B), because the hover probability was on average 88% larger than for landings from free-flight (Fig. 7). Thus, free-flying bumblebees landing in high sidewinds performed fewer hovering maneuvers than bumblebees landing after take-off, enabling them to fully compensate for the negative effects of the applied sidewinds. In contrast, bumblebees landing after take-off increased their landing time with increasing sidewind.

### Energetic costs of landing in a sidewind

In our experiments, the duration of landings directly after take-off increased with sidewind speed. This can negatively influence the foraging efficiency of bumblebees when they visit multiple flowers within a flower patch (Balfour et al., 2021). Moreover, bumblebees foraging in the fastest tested windspeed landed 70% less than in still air. Similar dynamics were observed in field and semi-field conditions, where the winds negatively impacted the foraging rate of honeybees (Hennessy et al., 2020, 2021; Pinzauti, 1986; Vicens and Bosch, 2000).

These combined results suggest that wind can negatively affect the fitness of individual insect pollinators and their colony (Riessberger and Crailsheim, 1997), and the efficacy of their pollination services (Tuell and Isaacs, 2010). Understanding these effects is crucial as insect pollinators support biodiversity (Ollerton et al., 2011) and global food production (Klein et al., 2007). This is even more pertinent with a predicted increase in windspeeds due to climate change in several areas of the world (Hosking et al., 2018). Future work in this direction could address the direct effect of winds on the colony fitness and pollination dynamics. Furthermore, testing bumblebees in higher wind conditions than was done here would allow for testing the limits of bumblebee foraging capabilities in windy conditions.

### Hypotheses for the control mechanisms of the flight response

**Fig. 9.**
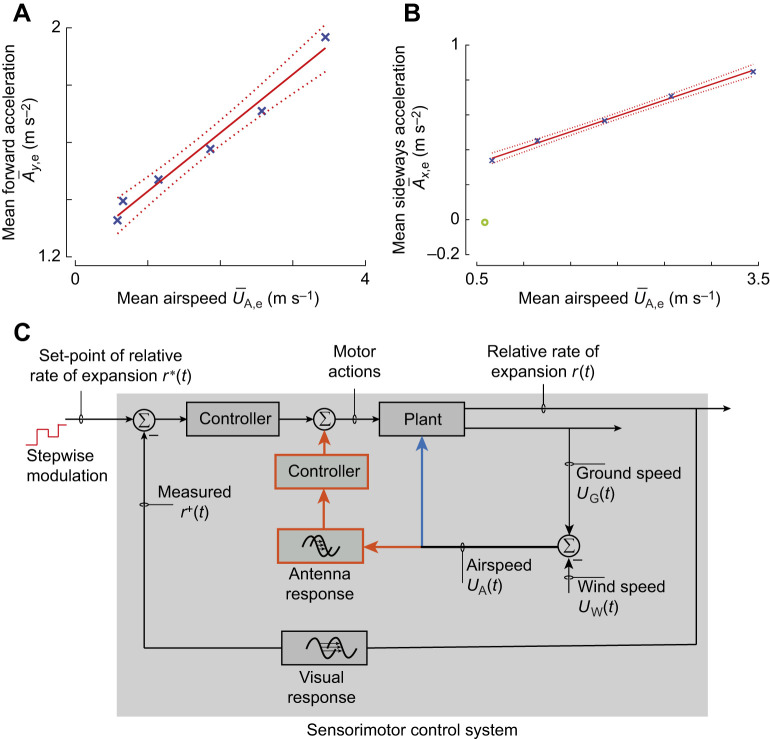
**Hypothetical multimodal sensorimotor control system for bumblebees landing in sidewind, with airspeed measurement integrated in the visual-feedback loop.** (A,B) During entry segments, the mean accelerations towards the landing platform (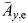; A) and with the wind (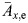; B) increase approximately linearly with mean airspeed 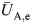, as estimated using linear mixed models (at the median values of covariates and averaged over landing types). Blue crosses depict the model results at each tested windspeed, and the solid and dotted red lines shows the linear fit and 95% confidence interval, respectively. (B) The zero-wind control case (green circle) was not included in the linear fit, because here no sideways acceleration is expected nor observed. (C) Proposed multimodal control model in landing bumblebees that explains the linear increase of the accelerations 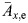 and 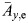 with airspeed 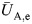. Wind-induced increase in airspeed directly acts on the plant (blue path), resulting in destabilizing aerodynamic forces and torques on the animal, and by increasing the aerodynamic control forces produced by the landing bumblebee. Bumblebees might rely on airflow sensory feedback from antennae (orange path) to stabilize their vision-based sensorimotor control loop. The fast positive feedback from the antennae can provide active damping that counteracts the unstable oscillations of the sidewind and visual feedback loops.

The increased control forces during transient responses in high wind can be evoked by: (1) direct wind-induced aerodynamic forces (blue arrow in Fig. 9), (2) active control through sensory feedback (antenna and visual responses in Fig. 9) or, more likely, (3) a combination of direct aerodynamic feedback and mechanosensory feedback (Fig. 9).

A bumblebee landing in a sidewind needs to produce an increased wingbeat-induced aerodynamic force, as it needs to produce forces not only for weight support, but also to cancel wind-induced parasite drag on the body and wings of the animal. Therefore, owing to the higher sidewind-induced steady state force production, a given control input from the bumblebee flying in a sidewind might lead to an increased control force output, which will consequently lead to higher aerodynamic forces and torques on the body. This increased control output can be regarded as a direct physical effect of sidewind on flight control actions (blue control path in Fig. 9C), and it thus effectively increases the control gain of the sensory-motor system. This increased gain could help to compensate for downwind drift, and may directly increase the animal's acceleration towards the landing platform, but may simultaneously also destabilize landing control (de Croon, 2016).

In addition, a bumblebee landing in a sidewind could also increase the transient accelerations towards the landing platform, by performing an increased active control action input in higher winds based on sensory feedback from an airspeed-measuring mechanosensory modality. Mechanoreceptors on the bumblebee's antennae are capable of detecting airspeed fast and precisely (Jakobi et al., 2018; Taylor and Krapp, 2007), and thus we suggest an antennae-based sensory-motor control feedback system for enhancing control actions in a sidewind (orange control path in Fig. 9C). The mechanical input to the antennae is a direct physical aerodynamic force, similar to the immediate aerodynamic feedback forces on the body. However, this sensory feedback loop involves a delay owing to the physical–chemical control path in the body. A similar sensory feedback system was suggested for free-flying *Drosophila* (Fuller et al., 2014).

To assess whether the combined direct mechanical feedback and sensory feedback loops explain the enlarged control responses in sidewinds, we tested how both 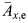 and 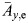 vary with the mean airspeed (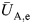) that bumblebees experienced during entry segments in different wind conditions. For both cases, a linear fit captured this interaction well (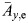 versus 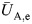: slope=0.204±0.015 s^−1^, *R*^2^=0.98; Fig. 9A; 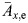 versus 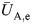: slope=0.181±0.006 s^−1^, *R*^2^=0.996; Fig. 9B). Thus, in both directions, the mean accelerations during entry segments increase approximately linearly with the airspeed induced by the sidewind. Note that here we assume that the control of forward and sideways flight accelerations is independent from each other, although these are inherently coupled. Further research is needed to quantify these non-linear control interactions.

The streamwise sideways accelerations that occur during the transient response phases are most likely the result of the wind-induced sideways drag force on the body and wings of the flying animal. An alternative explanation could be that in a sidewind, the bumblebee actively produces these sideways motions to estimate its distance to the landing surface, similar to how flies use sideways movements during landing (van Breugel et al., 2014). But such actively produced sideways movements could be produced in both the upwind and downwind direction. Because the sideways accelerations are consistently in the downwind direction, the sidewind-induced drag is most likely the driving factor here.

Aerodynamic drag forces on an object in air tend to scale quadratically with airspeed (Anderson, 1985), and thus for an exclusive direct mechanical feedback scenario (only the blue arrow in Fig. 9C), we would expect the relationship between sidewind and acceleration to be also quadratic. Thus, the observed linear relationship suggests that a (mechano-)sensory-based feedback is also at play (orange path in Fig. 9C). Therefore, we propose that a combination of wind-induced mechanical feedback and airspeed-based mechanosensory feedback improves the response time and stability of the vision-based control of landing bumblebees (both blue and orange pathways in Fig. 9C); additional work is needed to unravel the detailed controller design, and to estimate the relative contribution of the direct wind-induced feedback and sensory control feedback loops.

The question remains why bumblebees landing in a sidewind might use feedback from their wind-measuring sensory system to enhance their landing responses. The answer may lay in the results of the study on flight control in *Drosophila* (Fuller et al., 2014). As the neural processing time of information from antennae (∼20 ms) is much shorter than that of the visual system (∼50–100 ms), positive feedback from the antennal system can provide active damping to any vision-based regulator (Fuller et al., 2014). During sudden disturbances such as wind, active damping can reduce the oscillations of a relatively slow visual feedback loop. Active damping stabilizes the dynamics of insect locomotion in multiple other scenarios (Cheng and Deng, 2011; Cowan et al., 2006; Dyhr et al., 2013; Elzinga et al., 2012; Hedrick, 2011; Hedrick et al., 2009; Sun, 2014; Taylor et al., 2013), and thus mechanosensory control modulation in bumblebees landing in a sidewind might equally help stabilize their response actions.

The proposed combination of direct mechanical, visual and mechanosensory flight control systems may be commonly used by insects landing in windy conditions, and may also inspire the development of landing control strategies onboard robotic flight systems.

## Supplementary Material

10.1242/jexbio.245432_sup1 Supplementary information

## ECR Spotlight

This article has an associated ECR Spotlight interview with Pulkit Goyal.
