# Supplementary material for: Bumblebees compensate for the adverse effects of sidewind during visually guided landings
Source: J Exp Biol. 2024 Apr 22;227(8):jeb245432. doi: 10.1242/jeb.245432 (PMC11112349; doi:10.1242/jeb.245432)
Supplement: Supplementary information [file jexbio-227-245432-s1.pdf]

Table S1. Statistical analysis of landing frequency of bumblebees in different wind speeds. The post-hoc tests compare differences between the number of landings per hour N in different wind conditions (linear mixed model defined as: $N_{\text{takeoff},i,d,t}$ or $N_{\text{freeflight},i,d,t} \sim \mathcal{N}(\alpha_0 + \alpha_d + \alpha_t + \sum_{j=1}^{5} \beta_j \text{ WIND}_{j,i,d,t},\ \sigma^2)$).

Effect on $N_{\text{freeflight},i,d,t}$

Fixed effect	Estimate	Std error	t value	Pr(>|t|)
α	280.83	28.22	9.95	$8.34E-09$
β_1	−69.41	22.47	−3.09	0.0038
β_2	−94.72	22.68	−4.18	0.0002
β_3	−135.19	21.44	−6.31	$2.48E-07$
β_4	−165.55	21.79	−7.60	$4.31E-09$
β_5	−168.61	21.17	−7.97	$1.4E-09$

Post-hoc contrasts*	Estimate	Std error	z ratio	p value
0 - 1	69.41	22.55	3.08	0.057264
0 - 2	94.72	22.88	4.14	0.002631
0 - 3	135.19	21.48	6.29	$3.27E-06$
0 - 4	165.55	21.88	7.56	$5.58E-08$
0 - 5	168.61	21.27	7.93	$1.81E-08$
1 - 2	25.32	23.06	1.10	1
1 - 3	65.79	22.54	2.92	0.087547
1 - 4	96.15	22.24	4.32	0.001572
1 - 5	99.20	22.12	4.49	0.000929
2 - 3	40.47	22.98	1.76	1
2 - 4	70.83	22.28	3.18	0.043664
2 - 5	73.88	22.09	3.34	0.027328
3 - 4	30.36	22.09	1.37	1
3 - 5	33.41	21.57	1.55	1
4 - 5	3.05	21.14	0.14	1

*0, 1, 2, 3, 4, and 5 correspond to wind speeds 0, 0.3, 1.0, 1.8, 2.5, and 3.4 m s^{-1}, respectively.

(Continued on next page.)

Effect on $N_{\text{takeoff},i,d,t}$

Fixed effect	Estimate	Std error	t value	Pr(>|t|)
α	55.986	7.803	7.175	$4.39E-07$
β_1	-20.145	6.256	-3.220	0.002645
β_2	-23.300	6.332	-3.680	0.000717
β_3	-26.808	5.969	-4.491	$6.67E-05$
β_4	-33.637	6.071	-5.541	$2.51E-06$
β_5	-39.508	5.900	-6.696	$6.75E-08$

Post-hoc contrasts*	Estimate	Std error	z ratio	p value
0 - 1	20.14	6.27	3.21	0.03988
0 - 2	23.30	6.37	3.66	0.011189
0 - 3	26.81	5.97	4.49	0.000967
0 - 4	33.64	6.09	5.52	$3.64E-05$
0 - 5	39.51	5.92	6.68	$9.46E-07$
1 - 2	3.16	6.41	0.49	1
1 - 3	6.66	6.27	1.06	1
1 - 4	13.49	6.19	2.18	0.53075
1 - 5	19.36	6.15	3.15	0.047411
2 - 3	3.51	6.40	0.55	1
2 - 4	10.34	6.20	1.67	1
2 - 5	16.21	6.15	2.64	0.179731
3 - 4	6.83	6.15	1.11	1
3 - 5	12.70	6.00	2.12	0.610922
4 - 5	5.87	5.88	1.00	1

*0, 1, 2, 3, 4, and 5 correspond to wind speeds 0, 0.3, 1.0, 1.8, 2.5, and 3.4 m s^{-1}, respectively.

Table S2. Statistical analysis of mean relative-rate-of-expansion in different tested treatments (wind speeds and landing types) for average-landing-approach analysis. The data comprises of 19,421 landing approaches between 0.04 m $\leq y \leq$ 0.11 m, where y is the perpendicular distance to the platforms. Post-hoc tests compare differences between mean relative-rate-of-expansion observed in different tested conditions (linear mixed model defined as: $\bar{r}^*_{i,d,a,s} \sim \mathcal{N}(\alpha + \alpha_d + \alpha_a + \alpha_s + \sum_{j=1}^{5} \beta_j \ \mathrm{WIND}_{j,i,d,a,s} + \beta_6 \ \mathrm{fromTakeoff}_{i,d,a,s} + \sum_{j=7}^{11} \beta_j \ \mathrm{WIND}_{j,i,d,a,s} \times \mathrm{fromTakeoff}_{i,d,a,s}, \ \sigma^2))$.

Fixed effect	Estimate	Std error	t value	Pr(>|t|)
α	2.892	0.075	38.439	0.003595
β_1	0.003	0.049	0.062	0.950452
β_2	−0.059	0.051	−1.168	0.242655
β_3	−0.075	0.053	−1.416	0.156752
β_4	−0.092	0.056	−1.634	0.102325
β_5	0.011	0.057	0.196	0.844827
β_6	0.945	0.077	12.323	$9.62E-35$
β_7	−0.294	0.122	−2.412	0.015855
β_8	−0.584	0.128	−4.547	$5.47E-06$
β_9	−0.792	0.128	−6.185	$6.34E-10$
β_{10}	−1.122	0.139	−8.068	$7.62E-16$
β_{11}	−1.099	0.154	−7.120	$1.12E-12$

Post-hoc contrasts*	Estimate	Std error	z ratio	p value
0 - 1	0.291	0.112	2.600	0.13995
0 - 2	0.644	0.118	5.440	$7.98E-07$
0 - 3	0.867	0.117	7.431	$1.62E-12$
0 - 4	1.213	0.127	9.532	$2.31E-20$
0 - 5	1.088	0.143	7.585	$4.97E-13$
1 - 2	0.352	0.130	2.710	0.100911
1 - 3	0.575	0.128	4.479	0.000112
1 - 4	0.922	0.138	6.676	$3.68E-10$
1 - 5	0.796	0.153	5.202	$2.95E-06$
2 - 3	0.223	0.134	1.667	1
2 - 4	0.570	0.143	3.977	0.001046
2 - 5	0.444	0.158	2.816	0.072967
3 - 4	0.347	0.142	2.444	0.217702
3 - 5	0.221	0.156	1.413	1
4 - 5	−0.126	0.165	−0.764	1

*0, 1, 2, 3, 4, and 5 correspond to wind speeds 0, 0.3, 1.0, 1.8, 2.5, and 3.4 m s^{-1}, respectively.

*These post-hoc tests correspond to landings after a take-off. For landings from a free-flight, all comparisons among wind speeds were statistically insignificant.

Table S3. Statistical analysis of dependence of relative-rate-of-expansion set-points (r^*) on distance to the platform (y^*), different wind speeds and two starting conditions (take-off and free-flight). The data comprises of r^* and y^* for 12,338 constant-r segments in 9,097 landing manoeuvres. Post-hoc tests compare differences in $log(r^*)$ observed at mean $y^* = 0.185$ m in the presence of different wind speeds (factor $f = 1$) (linear mixed model defined as: $\log(r^*_{i,d,a,s}) \sim \mathcal{N}(\alpha + \alpha_d + \alpha_a + \alpha_s + \beta_1 \log(y^*_{i,d,a,s}) + \sum_{j=2}^{6} \beta_j \text{ WIND}_{j,i,d,a,s} + \beta_7 \text{ fromTakeoff}_{i,d,a,s} + \beta_8 \log(y_{i,d,a,s}) \times \text{fromTakeoff}_{i,d,a,s}, \sigma^2))$.

Fixed effect	Estimate	Std error	t value	Pr(>|t|)
α	−0.539	0.018	−30.237	1.77E − 89
β_1	−0.727	0.008	−89.275	0
β_2	0.004	0.011	0.361	0.718181
β_3	0.009	0.012	0.769	0.441659
β_4	0.041	0.012	3.302	0.000966
β_5	0.076	0.013	5.691	1.31E − 08
β_6	0.148	0.015	9.727	3E − 22
β_7	−0.309	0.035	−8.849	1.01E − 18
β_8	−0.234	0.019	−12.238	3.1E − 34

Post-hoc constrasts* in $log(r^*)$ at mean $y^* = 0.185$ m	Estimate	Std error	z ratio	p value
0 - 1	−0.004	0.011	−0.361	1
0 - 2	−0.009	0.012	−0.769	1
0 - 3	−0.041	0.012	−3.302	0.01442
0 - 4	−0.076	0.013	−5.691	1.9E − 07
0 - 5	−0.148	0.015	−9.727	3.47E − 21
1 - 2	−0.005	0.013	−0.392	1
1 - 3	−0.037	0.013	−2.748	0.089829
1 - 4	−0.072	0.014	−5.051	6.6E − 06
1 - 5	−0.144	0.016	−8.984	3.9E − 18
2 - 3	−0.032	0.014	−2.274	0.34472
2 - 4	−0.067	0.015	−4.503	0.000101
2 - 5	−0.139	0.017	−8.394	7.04E − 16
3 - 4	−0.035	0.015	−2.299	0.322887
3 - 5	−0.107	0.017	−6.356	3.11E − 09
4 - 5	−0.072	0.018	−4.121	0.000565

*0, 1, 2, 3, 4, and 5 correspond to wind speeds 0, 0.3, 1.0, 1.8, 2.5, and 3.4 m s^{-1}, respectively.

*The results are averaged over landing types because wind speeds had similar effect on both landing types.

Journal of Experimental Biology • Supplementary information

Table S4. Statistical analysis of how during entry segments bumblebees modulate the expansion-acceleration ($\dot{r}_e$), mean acceleration towards the landing platform ($\overline{A}_{y,e}$), and mean sideways acceleration ($A_{x,e}$) with wind speed. The data comprises 4,221 entry segments in 4,038 landing maneuvers. The linear mixed models are defined as $\log(\dot{r}_{e\ i,d,a,s})$ or $\log(A_{y,e\ i,d,a,s}) \sim \mathcal{N}(\alpha + \alpha_d + \alpha_a + \alpha_s + \beta_1 \log(y_{0\ i,d,a,s}) + \sum_{j=2}^{6} \beta_j \text{WIND}_{j,i,d,a,s} + \beta_7 \text{fromTakeoff}_{i,d,a,s} + \beta_8 \log(\Delta r_{e\ i,d,a,s}) + \beta_9 \log(r^*_{i,d,a,s}) + \beta_{10} \log(\Delta r_{e\ i,d,a,s}) \times \log(y_{0\ i,d,a,s}) + \beta_{11} \log(\Delta r_{e\ i,d,a,s}) \times \log(r^*_{i,d,a,s}), \sigma^2)$ and $\overline{A}_{x,e\ i,d,a,s} \sim \mathcal{N}(\alpha + \alpha_d + \alpha_a + \alpha_s + \beta_1 x_{0\ i,d,a,s} + \sum_{j=2}^{6} \beta_j \text{WIND}_{j,i,d,a,s} + \beta_7 \text{fromTakeoff}_{i,d,a,s} + \sum_{j=8}^{12} \beta_j \text{WIND}_{j,i,d,a,s} \times \text{fromTakeoff}_{i,d,a,s} + \beta_{13} x_{0\ i,d,a,s} \times \text{fromTakeoff}_{i,d,a,s} + \sum_{j=14}^{18} \beta_j \text{WIND}_{j,i,d,a,s} \times x_{0\ i,d,a,s} + \sum_{j=19}^{23} \beta_j \text{WIND}_{j,i,d,a,s} \times x_{0\ i,d,a,s} \times \text{fromTakeoff}_{i,d,a,s}, \sigma^2)$.

Effect on $\dot{r}_e$

Fixed effect	Estimate	Std error	t value	Pr(>|t|)
α	1.466	0.030	48.337	$6.77E-33$
β_1	−0.289	0.022	−12.896	$2.41E-37$
β_2	0.032	0.012	2.606	0.009182
β_3	0.049	0.013	3.782	0.000158
β_4	0.111	0.013	8.253	$2.04E-16$
β_5	0.152	0.014	10.678	$2.78E-26$
β_6	0.235	0.016	14.488	$1.86E-46$
β_7	−0.035	0.012	−3.055	0.002262
β_8	0.034	0.036	0.941	0.346987
β_9	0.243	0.021	11.354	$1.9E-29$
β_{10}	−0.362	0.025	−14.681	$1.26E-47$
β_{11}	−0.069	0.015	−4.770	$1.9E-06$

Effect on $\overline{A}_{y,e}$

Fixed effect	Estimate	Std error	t value	Pr(>|t|)
α	1.973	0.058	34.030	$9.27E-56$
β_1	0.529	0.043	12.425	$8.04E-35$
β_2	0.049	0.023	2.113	0.03465
β_3	0.102	0.025	4.140	$3.57E-05$
β_4	0.172	0.025	6.757	$1.63E-11$
β_5	0.253	0.027	9.445	$6.29E-21$
β_6	0.393	0.030	12.897	$3.32E-37$
β_7	−0.138	0.022	−6.272	$4.01E-10$
β_8	0.590	0.069	8.567	$1.49E-17$
β_9	−1.632	0.044	−36.770	$2.5E-255$
β_{10}	−0.679	0.047	−14.490	$1.95E-46$
β_{11}	−0.103	0.027	−3.754	0.000176

(Continued on next page.)

Effect on $\overline{\boldsymbol{A}}_{\boldsymbol{x},\mathrm{e}}$

Fixed effect	Estimate	Std error	t value	Pr(>|t|)
α	$3.03E-01$	$7.96E-02$	3.807	0.010701
β_1	$-1.80E+00$	$5.07E-01$	−3.542	0.000402
β_2	$2.77E-01$	$9.12E-02$	3.038	0.002394
β_3	$4.78E-01$	$9.84E-02$	4.856	$1.24E-06$
β_4	$3.95E-01$	$1.03E-01$	3.841	0.000124
β_5	$5.67E-01$	$1.10E-01$	5.156	$2.64E-07$
β_6	$9.33E-01$	$1.21E-01$	7.736	$1.27E-14$
β_7	$-2.75E-01$	$1.28E-01$	−2.16	0.030855
β_8	$1.33E-01$	$2.20E-01$	0.607	0.544111
β_9	$4.34E-03$	$2.37E-01$	0.018	0.985417
β_{10}	$2.20E-01$	$2.38E-01$	0.926	0.354675
β_{11}	$1.79E-01$	$2.47E-01$	0.727	0.467413
β_{12}	$-3.08E-01$	$3.09E-01$	−0.995	0.319756
β_{13}	$-7.55E+00$	$1.81E+00$	−4.178	$3.00E-05$
β_{14}	$-2.88E-01$	$7.97E-01$	−0.362	0.71762
β_{15}	$-7.31E-01$	$8.16E-01$	−0.896	0.37011
β_{16}	$3.58E-01$	$8.73E-01$	0.41	0.681851
β_{17}	$-9.58E-01$	$9.25E-01$	−1.036	0.300084
β_{18}	$-3.37E+00$	$9.00E-01$	−3.747	0.000181
β_{19}	$1.26E+00$	$2.85E+00$	0.441	0.659017
β_{20}	$7.23E-01$	$3.02E+00$	0.24	0.810546
β_{21}	$4.03E+00$	$2.89E+00$	1.396	0.162687
β_{22}	$6.04E+00$	$3.23E+00$	1.873	0.061106
β_{23}	$1.19E+01$	$3.98E+00$	2.992	0.002792

Table S5. Statistical analysis of how often a bumblebee exhibits a hover phase ($V < 0.05$ m s^{-1}). It depends upon the wind speed, the landing type (landing from free-flight or take-off) and distance to the surface y. There are six wind speeds (m s^{-1}: $w_0 = 0$, $w_1 = 0.3$, $w_2 = 1.0$, $w_3 = 1.8$, $w_4 = 2.5$, $w_5 = 3.4$) and four distance regions (y_1(0.05 m $< y \leq$ 0.10 m), y_2(0.10 m $< y \leq$ 0.15 m), y_3(0.15 m $< y \leq$ 0.20 m) and y_4(0.20 m $< y \leq$ 0.25 m)) (linear mixed model defined as: $P \sim$ yRegion + wind + hasTakeoff + hasTakeoff × wind + yRegion × hasTakeoff + wind × yRegion + (1|day) + (1|approach) + (1|landingSide), estimate of effects is in *logit* scale).

Fixed effect	Estimate	Std error	z value	Pr(>|t|)
Intercept	−0.51	0.11	−4.66	3.09E − 06
y_2	−1.14	0.06	−19.25	1.45E − 82
y_3	−1.74	0.07	−25.45	6.3E − 143
y_4	−2.06	0.07	−27.69	8.7E − 169
w_1	0.12	0.06	1.99	0.046167
w_2	0.37	0.06	6.15	7.96E − 10
w_3	0.73	0.06	11.85	2.04E − 32
w_4	0.87	0.07	13.13	2.12E − 39
w_5	1.22	0.07	17.06	2.75E − 65
hasTakeoff	−0.14	0.07	−1.97	0.049158
w_1 : hasTakeoff	0.22	0.10	2.15	0.031574
w_2 : hasTakeoff	0.40	0.10	3.92	8.72E − 05
w_3 : hasTakeoff	0.61	0.10	6.23	4.61E − 10
w_4 : hasTakeoff	0.76	0.11	7.20	6E − 13
w_5 : hasTakeoff	0.71	0.12	6.05	1.46E − 09
y_2 : hasTakeoff	−0.78	0.08	−10.22	1.59E − 24
y_3 : hasTakeoff	−0.83	0.09	−9.27	1.88E − 20
y_4 : hasTakeoff	−0.63	0.09	−6.69	2.17E − 11
$y_2 : w_1$	−0.06	0.09	−0.72	0.471313
$y_3 : w_1$	−0.20	0.11	−1.91	0.056157
$y_4 : w_1$	−0.18	0.11	−1.59	0.111079
$y_2 : w_2$	−0.06	0.09	−0.63	0.531825
$y_3 : w_2$	−0.10	0.10	−0.93	0.35172
$y_4 : w_2$	−0.06	0.11	−0.55	0.58055
$y_2 : w_3$	−0.13	0.09	−1.42	0.156609
$y_3 : w_3$	−0.31	0.10	−3.00	0.00269
$y_4 : w_3$	−0.37	0.11	−3.30	0.000952
$y_2 : w_4$	−0.12	0.10	−1.23	0.220355
$y_3 : w_4$	−0.22	0.11	−2.10	0.035948
$y_4 : w_4$	−0.28	0.11	−2.43	0.01494
$y_2 : w_5$	−0.33	0.10	−3.28	0.00104
$y_3 : w_5$	−0.45	0.11	−4.04	5.34E − 05
$y_4 : w_5$	−0.47	0.12	−3.92	8.69E − 05

Journal of Experimental Biology • Supplementary information

Table S6. Statistical analysis of how travel time of landing bumblebees (Δt) depends on the wind speeds and two landing types (landing after a take-off or from a free-flight). The data comprises of travel time Δt of bumblebees that they took to cover 0.2 m approach distance y (from $y = 0.25$ m to $y = 0.05$ m from the landing platform) in all landing maneuvers that started beyond $y = 0.25$ m. (linear mixed model defined as: $\Delta t_{i,d,s} \sim \mathcal{N}(\alpha + \alpha_d + \alpha_s + \sum_{j=1}^{5} \beta_j \ \mathrm{WIND}_{j,i,d,s} + \beta_6 \ \mathrm{fromTakeoff}_{i,d,a,s} + \sum_{j=7}^{11} \beta_j \ \mathrm{WIND}_{j,i,d,s} \times \mathrm{fromTakeoff}_{i,d,s})$.

Fixed effect	Estimate	Std error	t value	Pr(>|t|)
α	0.723	0.028	25.742	0.00903
β_1	−0.026	0.011	−2.389	0.016917
β_2	0.005	0.011	0.435	0.663802
β_3	0.006	0.011	0.561	0.574797
β_4	0.032	0.012	2.607	0.00915
β_5	−0.030	0.013	−2.407	0.016084
β_6	−0.175	0.014	−12.217	$3.81E-34$
β_7	0.021	0.023	0.930	0.352305
β_8	0.065	0.024	2.673	0.007518
β_9	0.121	0.024	5.019	$5.26E-07$
β_{10}	0.133	0.027	4.975	$6.6E-07$
β_{11}	0.219	0.030	7.250	$4.39E-13$

Journal of Experimental Biology • Supplementary information
